# Assessing Phylogenetic Relationships among Galliformes: A Multigene Phylogeny with Expanded Taxon Sampling in Phasianidae

**DOI:** 10.1371/journal.pone.0064312

**Published:** 2013-05-31

**Authors:** Ning Wang, Rebecca T. Kimball, Edward L. Braun, Bin Liang, Zhengwang Zhang

**Affiliations:** 1 Key Laboratory for Biodiversity Sciences and Ecological Engineering of Ministry of Education, College of Life Sciences, Beijing Normal University, Beijing, China; 2 Department of Biology, University of Florida, Gainesville, Florida, United States of America; 3 Key Laboratory for Tropical Plant and Animal Ecology of Ministry of Education, College of Life Sciences, Hainan Normal University, Haikou, China; BiK-F Biodiversity and Climate Research Center, Germany

## Abstract

Galliform birds (relatives of the chicken and turkey) have attracted substantial attention due to their importance to society and value as model systems. This makes understanding the evolutionary history of Galliformes, especially the species-rich family Phasianidae, particularly interesting and important for comparative studies in this group. Previous studies have differed in their conclusions regarding galliform phylogeny. Some of these studies have suggested that specific clades within this order underwent rapid radiations, potentially leading to the observed difficulty in resolving their phylogenetic relationships. Here we presented analyses of six nuclear intron sequences and two mitochondrial regions, an amount of sequence data larger than many previous studies, and expanded taxon sampling by collecting data from 88 galliform species and four anseriform outgroups. Our results corroborated recent studies describing relationships among the major families, and provided further evidence that the traditional division of the largest family, the Phasianidae into two major groups (“pheasants” and “partridges”) is not valid. Within the Phasianidae, relationships among many genera have varied among studies and there has been little consensus for the placement of many taxa. Using this large dataset, with substantial sampling within the Phasianidae, we obtained strong bootstrap support to confirm some previously hypothesized relationships and we were able to exclude others. In addition, we added the first nuclear sequence data for the partridge and quail genera *Ammoperdix*, *Caloperdix*, *Excalfactoria*, and *Margaroperdix*, placing these taxa in the galliform tree of life with confidence. Despite the novel insights obtained by combining increased sampling of taxa and loci, our results suggest that additional data collection will be necessary to solve the remaining uncertainties.

## Introduction

The avian order Galliformes (landfowl) contains about 290 species [1], many of which (e.g., chicken, turkey and peacock) are closely related to human society. Some of these birds are also important model systems in areas as diverse as development [2], disease transmission [3], and sexual selection [4]. Thus, a well-resolved galliform phylogeny is necessary to address a wide range of questions such as the geographic origin of certain lineages [5], the possible transmission and evolution of avian pathogens [3], and the evolution of sexual traits [4], [6]–[9]. Finally, approximately 25% of galliform species worldwide are threatened (critically endangered, endangered, or vulnerable) based upon the IUCN red list [10]. This includes 11 galliform species found in China of which ten were included in our phylogeny. There is substantial interest in using phylogenetic information to inform conservation priorities [11] and previous efforts to understand the use of phylogenetic information to establish conservation priorities have used galliforms as a model system [12], making a well-resolved galliform phylogeny even more critical.

**Figure 1:**
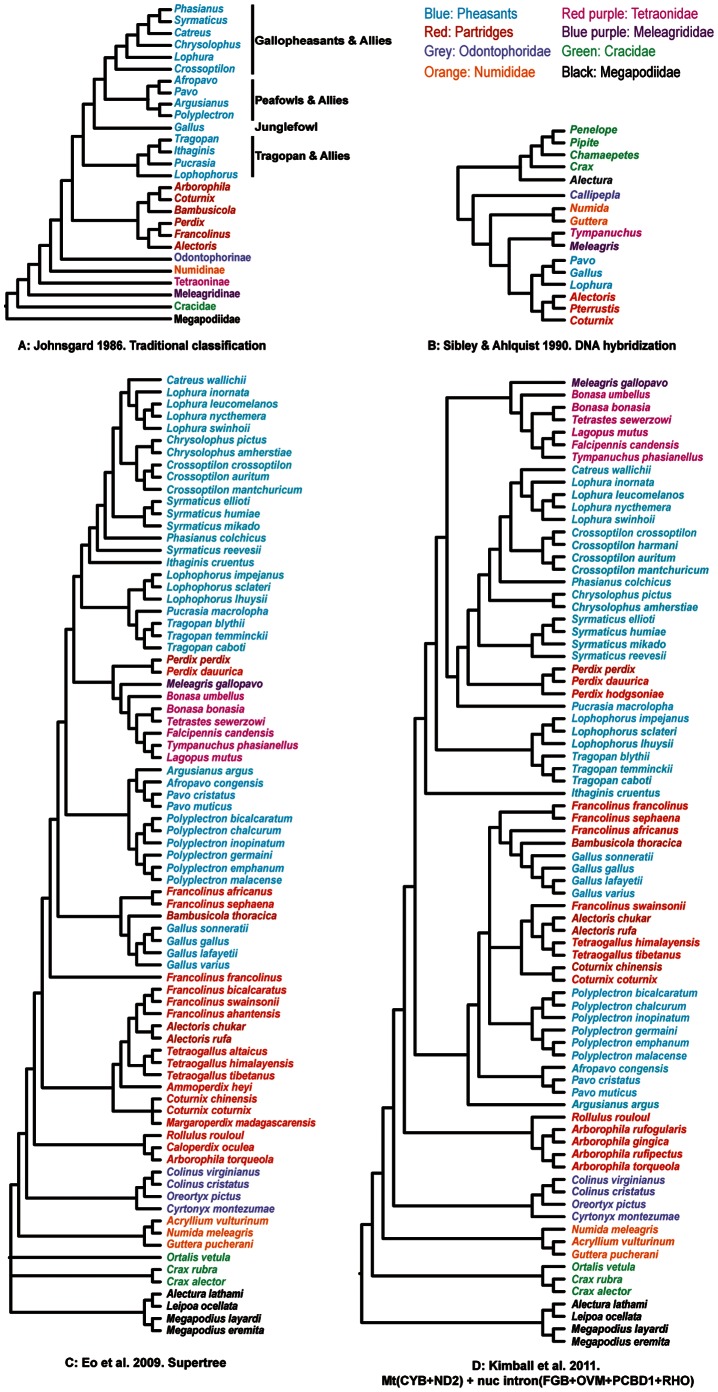
Representative prior hypotheses of Galliformes phylogeny. A more complete review of hypotheses can be found in Figure S1.

The sister group relationship between Galliformes and Anseriformes in the avian tree of life is very strongly supported [13], although relationships among the lineages within Galliformes are still controversial [4]–[5], [14]–[17](e.g., Figure 1 and Figure S1). Traditionally, Galliformes are divided into seven families: Megapodiidae (megapodes), Cracidae (guans, chachalacas, and currasows), Odontophoridae (New World quail), Numididae (guineafowls), Phasianidae (pheasants, partridges and allies), Meleagrididae (turkeys), and Tetraonidae (grouse and ptarmigan) [1], [18]. In traditional classifications, Phasianidae are sometimes split into two tribes (sometimes elevated to subfamilies), the Phasianini (pheasants) and Perdicini (partridges and Old World quail) [19], although the exact circumscription of these groups differs among authors. However, more recent studies argue against these classifications because they nest the turkeys, grouse, and ptarmigan within Phasianidae (Figure S1). Moreover, recent studies intermix the traditional pheasants and partridges, regardless of how those groups are circumscribed [5], [14]. Thus, for practical reasons, we will divide Galliformes into five families (placing Meleagrididae and Tetraonidae within Phasianidae) and subdivide the Phasianidae into turkeys, grouse, pheasants, and partridges for discussion. When referring to pheasants or partridges, we use Johnsgard’s [20], [21] circumscriptions (see Table S1).

Various types of data have been used to elucidate relationships within Galliformes, with researchers in the early to mid 1900 s using traits such as tail molt patterns [22] or anatomical traits [23]. Although those studies do not use cladistic methods, their classifications are used to develop “traditional” ideas regarding relationships within Galliformes [20], [24] (Figure 1A). Later studies include a variety of biochemical and molecular methods, ranging from immunological comparisons of protein and allozyme analyses [25]–[27], protein sequence data [26], [28], to DNA-DNA hybridization and restriction site analyses [18], [29]. Sibley and Ahlquist [18], by combining the results of DNA-DNA hybridization analyses with other lines of evidence available at that time, hypothesize that the turkeys and grouse nest within the Phasianidae (Figure 1B). More recent analyses use sequence data [17] (both mitochondrial and nuclear), cladistic analyses of morphological and behavioral traits [16] (hereafter M/B traits), transposable element (TE) insertions [30], [31], and combinations of various subsets of these data [5] to build galliform phylogenies. Although there are varying levels of disagreement among those more recent studies, it is possible to use supertree methods to summarize their results and visualize the best-supported clades in them [7], [32] (e.g., Figure 1C). These topologies can be viewed as informal priors that can be used to evaluate novel data, although it is clear that there are substantial differences among these studies. Thus, in spite of intensive study, a well-resolved and supported phylogeny representing a large diversity of gamebirds remains to be realized.

The remaining controversies regarding the branching order of Galliformes, especially within the Phasianidae, limit our ability to examine their evolution from many different perspectives. This includes studies as wide ranging as comparative morphology [8], [17], behavioral and ecological comparisons for these taxa [6], [7], and assessments of conservation priorities [11]. Thus, additional studies focused on this group are warranted. Here we focus on extending mitochondrial DNA (Mt) and nuclear intron dataset to improve taxon sampling, particularly within the Phasianidae, while limiting the amount of missing data. Using this data matrix we 1) placed our results in the context of a historical review of previous estimates of galliform phylogeny; 2) added taxa for which the molecular data were limited or absent; 3) attempted to resolve groups that were controversial in previous studies; and 4) discussed the classification of Galliformes based on our best estimate of their phylogeny.

## Materials and Methods

### Ethics Statement

The majority of samples for this study were part of existing tissue collections and thus were not collected for this study. For the remaining samples, permission to collect the samples was granted by the Management Bureau of local national reserves (Dongzhai National Nature Reserve in Henan Province and Shennongjia National Nature Reserve in Hubei Province) or the managers of Beijing Breeding Center for Endangered Animals. Tissue samples were collected from birds that were dead naturally in the field and blood samples were collected gently from brachial veins of captured birds that were released afterwards. All procedures involving the handling of birds were approved by the Institutional Animal Care and Use Committee in Beijing Normal University.

### Data Collection

We sampled 92 species, four of which were outgroups (from Anseriformes, the sister order of Galliformes) (Table S1). While data for some species were taken from Genbank, most data (∼80%) have not been published previously.

DNA was extracted from blood or muscle tissue using the TIANamp Genomic DNA Kit (TIANGEN Biotech, China) or the PUREGENE**®** DNA Purification Kit (Gentra Systems). We amplified two Mt genes (ND2 and 12S) and six nuclear intron sequences (CLTC, CLTCL1, EEF2, FGB, SERPINB14, and RHO) using a combination of published [33], [34] and unpublished primers (Table S2). Loci were initially amplified using a single annealing temperature optimized for each locus, but later samples were amplified using a single touchdown PCR protocol that was able to successfully amplify all loci (the annealing temperature is from 58°C to 48°C, being reduced 0.5°C per cycle followed by 20 additional cycles at 48°C). All PCR products were examined for size on 1% agarose gel and purified using a PCR purification Kit (QIAGEN) or by PEG:NaCl (20%:2.5 M) precipitation. Samples were sequenced in both directions, using either ABI BigDye® Terminator v.3.1 on an ABI Prism™ 3100-Avant genetic analyzer (PE Applied Biosystems) or by the Beijing Genomic Institute. Sequences were assembled using either Sequencher™ 4.1 (Gene Codes Corp.) or MEGA 4.0 [35]. The novel sequences collected in this study have been deposited in Genbank (KC749446–KC749475, KC749573, KC749617–KC749662, KC785603-KC785731, KC749686–KC749759, KC749858–KC749906, KC791426, KC778809-KC778981, Table S3).

### Phylogenetic Analyses

#### Sequence alignment

Different alignment methods can have a major impact on phylogenetic estimation [36], [37]. To determine whether alignment influenced our conclusions, we aligned individual locus using three automated alignment programs, Mafft v.6.717 [38], Tcoffee [39], and Muscle [40]. We then combined alignments generated by the same program into three concatenated data matrices (only Mt genes, only nuclear introns, and all sequence data) using combine-0.9 (written by ELB). To assess the impact of the different sequence alignment programs, we conducted unpartitioned and partitioned maximum likelihood (ML) analyses in RAxML 7.2.6 [41] using the GTR+Γ model and ten randomized starting trees (all partitioned analyses were partitioned either by locus or by defining each gene type as a partition: Mt genes vs. Nuclear introns). Then we generated a neighbor-joining (NJ) tree of the Robinson-Foulds (RF) distances [42] among these trees to visualize their differences. Although the alignments given by the three programs differed in the positions and lengths of gaps, all combined data matrices using the same data type constructed trees that were very similar to each other (RF distances among trees that resulted from analyses using different alignments were similar to the RF distances among trees that resulted from different analyses using the same alignment, Figure S2), suggesting that sequence alignment had little impact upon tree reconstruction. Thus, we conducted the remaining analyses using the alignment generated by Mafft v.6.717.

#### Data analyses

Phylogenetic analyses were conducted by using the ML criterion and Bayesian Markov Chain Monte Carlo (MCMC) inference. The best fitting models for individual locus and the concatenated dataset were established in Modeltest 3.7 [43] using the second-order variant of the Akaike information Criterion (AIC_c_). For the concatenated dataset, we conducted ML analysis in PAUP*4.0b10 [44] using the best fitting model (GTR+Γ+I) in addition to the analyses conducted using the GTR+Γ model in RAxML (as described above in the material on sequence alignment). RAxML and PAUP* identified the same optimal tree topology; since RAxML is more computationally efficient than PAUP*, all other ML analyses were conducted in RAxML using the GTR+Γ model and 10 randomized starting trees. We compared the fit of the GTR+Γ model with and without partitioning by using the AIC_c_ based on the likelihood scores, using the numbers of free parameters reported by RAxML and treating the number of variable characters as the sample size. We also used RAxML with GTR+Γ to conduct ML bootstrap analyses with 500 replicates [45] with and without partitioning.

MrBayes3.1 [46] was used to conduct Bayesian MCMC analyses. Each locus was assigned the best model based on the AIC_c_ (or the closest available model implemented in MrBayes). We conducted two runs simultaneously with each having one cold chain and three heated chains and ran the analyses for 10 million generations, sampling every 100 generations. The standard deviation of split frequencies between the two runs were below 0.01, which indicated a convergence upon a specific topology. The potential scale reduction factor (PSRF [47]) was also used as a diagnostic to examine convergence. Convergence is suggested if the PSRF approaches 1, as they did for our analyses. Moreover, the effective sample size (ESS) values of various parameters were all greater than 200 based upon inspection using Tracer v1.4 [48], which suggested a sufficient sampling in the MCMC runs. Finally, our analyses appeared to converge based upon analyses using AWTY [49] (Figure S3). We deleted the first 25% of trees as burn-in and used the rest to generate the consensus tree.

#### Gene tree - species tree analyses

We also estimated the species tree from individual gene trees, in addition to using concatenation. We used both NJst [50] and STAR [51] on the Species Tree Webserver [52]. To accommodate uncertainty in the gene tree estimates, we performed 500 bootstrap replicates on each locus (combining the two mitochondrial regions as a single locus) using the GTR+Γ model in RAxML. STAR requires rooted gene trees with a single outgroup sequence. Therefore, we used a single anseriform (the Southern screamer *Chauna torquata*) as the outgroup. Since one locus, SERPINB14, lacked outgroup sequences, this locus was not included in the STAR analysis. We conducted two NJst analyses, one using all loci and a second excluding SERPINB14 (to allow a direct comparison to the results from STAR).

#### Testing the robustness of phylogenetic inference

We used several methods to test the robustness of our estimate of the tree topology. First, the Bayesian posterior probability and the ML bootstrap values were used to assess the support of each clade. Second, gene jackknifing was used to determine whether there was a single gene that had a large impact on the tree topology by excluding one locus at a time and using the remaining data for analyses in RAxML. Moreover, similar analyses were conducted by excluding either Mt genes (leaving only nuclear introns) or nuclear introns (leaving only Mt genes) to test the contribution of different type of DNA markers to the overall ML tree topology.

We also explored whether substitutional saturation or base compositional heterogeneity might have affected our conclusions. We used the *I*_ss_ metric calculated by DAMBE [53] to test for saturation (incorporating the estimated proportion of invariant sites) and the χ^2^ test implemented in PAUP* to examine the homogeneity of base frequencies across taxa for each locus. Finally, we conducted RY coding (changing purines [A and G] to R and pyrimidines [C and T] to Y) because many previous analyses indicate that this strategy can reduce the influence of substitutional saturation and base compositional heterogeneity. This was accomplished by using combine-perl (written by ELB) and then reestimating the phylogeny for both unpartitioned and the partitioned analyses in RAxML as described above.

## Results

### Patterns of Sequence Evolution for the Combined Dataset

The concatenated data matrix aligned by Mafft was 7147 bp in length and it had 69% variable sites and 49.4% informative sites. There were 2119 bp of Mt DNA sequence with 55% variable sites and 47.8% informative sites, while the remaining 5028 sites were from nuclear intron sequences that had 61.6% variable sites and 50% informative sites. Thus, the nuclear intron sequences had a greater percentage of variable sites than Mt genes. The consistency index (CI) and the retention index (RI) of the nuclear introns were higher than that of the Mt genes (Table 1). The shape parameter (α) of the Γ distribution describing among-sites rate variation, which was included in the best-fit models for all partitions except SERPINB14, was lower for the two Mt genes than in the nuclear introns, indicating that the Mt regions had greater among-site rate heterogeneity (Table 1). The results were consistent with the hypothesis that the nuclear introns exhibit less complex patterns of molecular evolution relative to Mt genes [15], [54].

**Table 1:** Characteristics of the two Mt regions and six nuclear loci.

Locus	Length	Informative	AIC_c_	CI	RI	Gamma (Alpha)
**Concatenated**	**7147**	**3530**	**GTR+I+Γ**	**0.388**	**0.716**	**0.5924**
12S	1075	412	GTR+I+Γ	0.247	0.616	0.8079
ND2	1044	602	GTR+I+Γ	0.212	0.576	0.7148
**Combined Mt**	**2119**	**1014**	**GTR+I+Γ**	**0.224**	**0.589**	**0.7449**
CLTC	816	407	HKY+I+Γ	0.605	0.846	1.6812
CLTCL1	605	285	TVM+Γ	0.597	0.821	2.5321
RHO	1152	682	TVM+Γ	0.556	0.850	2.0432
FGB	675	402	K81uf+Γ	0.623	0.849	3.4138
EEF2	1125	412	TVM+I+Γ	0.515	0.791	1.6382
SERPINB14	655	329	GTR	0.706	0.860	N/A
**Combined Nuclear**	**5028**	**2516**	**TVM+Γ**	**0.576**	**0.828**	**1.6631**

CI: consistency index; RI: Retention Index.

### Higher Level Relationships among Galliform Birds

**Figure 2:**
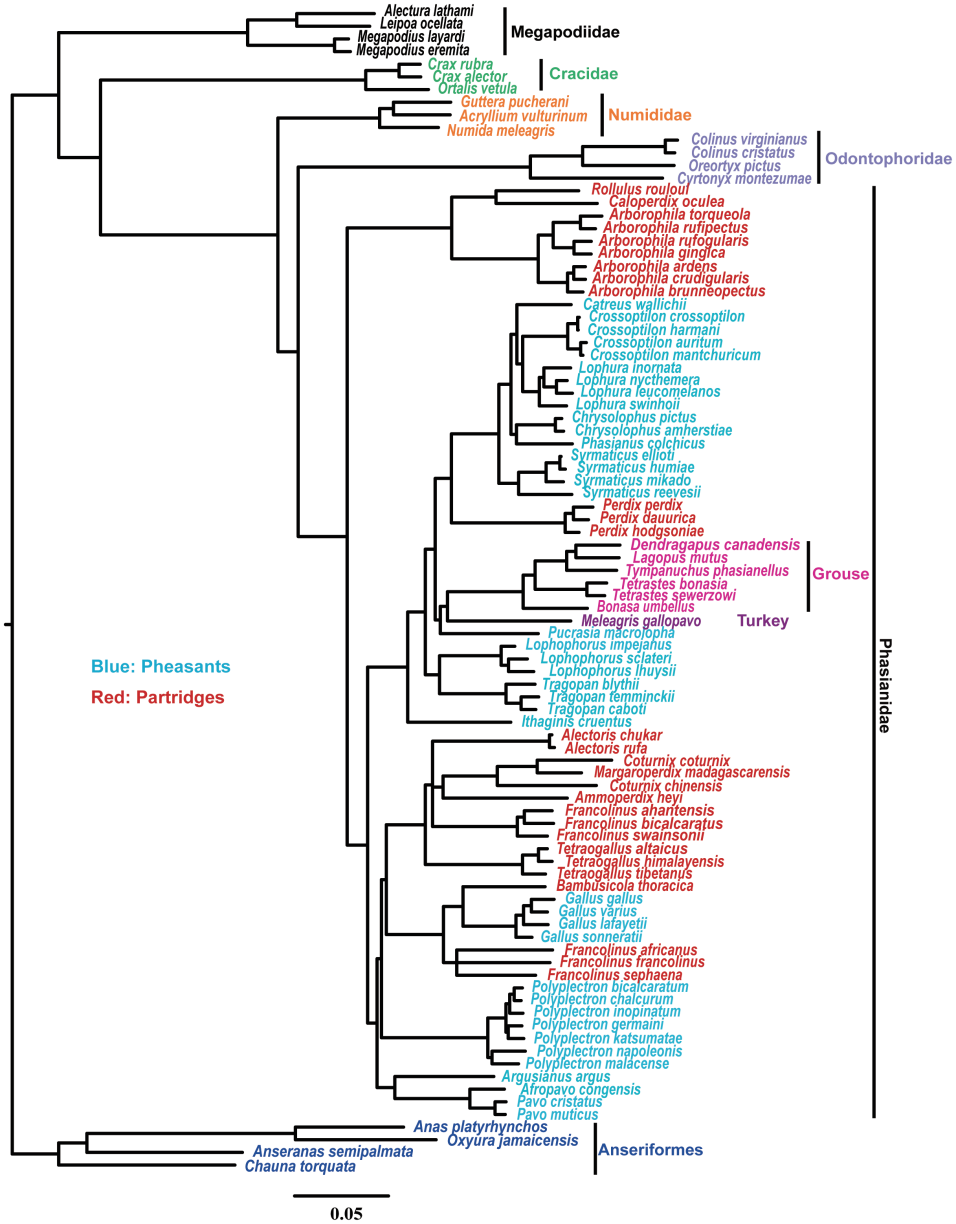
Estimate of Galliformes phylogeny based upon Bayesian MCMC analysis of the complete data matrix. Groups of taxa are indicated using the same color coding as Figure 1. Support values for this analysis are shown in Figure 3.

**Figure 3:**
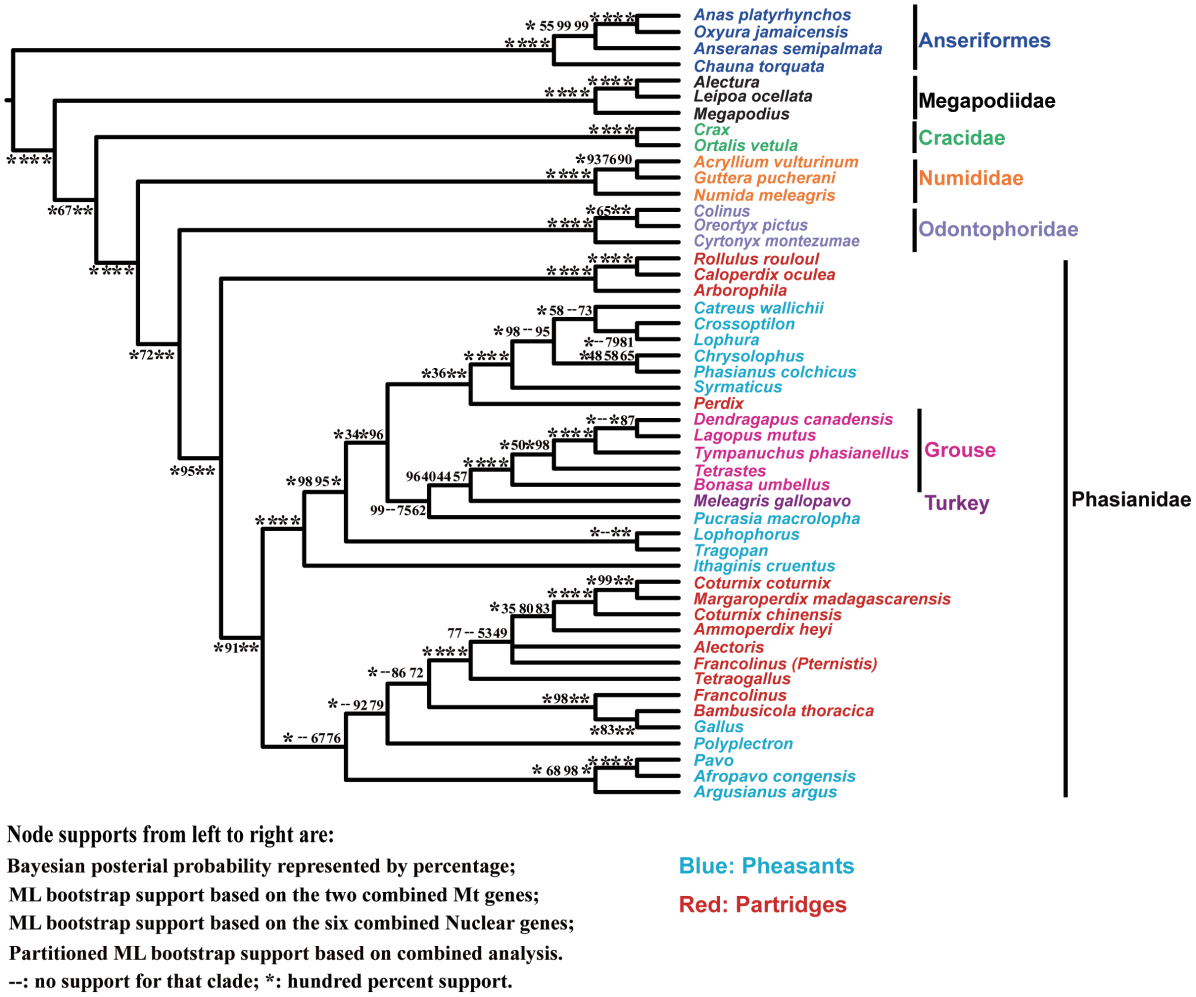
Consensus tree with support from concatenated analyses. Monophyletic genera with more than one species are collapsed into a single branch. Groups of taxa are indicated using the same color coding as Figure 1.

The topologies of the partitioned ML trees, whether using two-partitions (Mt and nuclear) or eight partitions, were identical, although there were differences between the unpartitioned and the partitioned ML trees (Figure S4 and the Treefile S1). The partitioned model (i.e., partitioned by locus) fit the data better than the unpartitioned model based upon the AIC_c_ (185769.1 vs. 190842.9). Moreover, the Bayesian consensus tree had almost the same tree topology as that given by the partitioned ML analyses (Figures 2 and 3, Figure S4 and the Treefile S1). These analyses confirmed there are five major clades within Galliformes that correspond to five of the traditional families (Figure 2). The first divergence within Galliformes was between Megapodiidae and all other taxa, and the remaining families branched successively after that in the order Cracidae, Numididae, Odontophoridae, and Phasianidae. The traditional Meleagrididae and Tetraonidae (hereafter referred to as turkey and grouse) nested within the Phasianidae and were sister to each other.

The most species-rich family within Galliformes, Phasianidae, recovered robust relationships among genera in the Bayesian analysis (Figure 2). In general, there were three major clades within Phasianidae. The earliest divergence was between Arborophilinae *sensu* Crowe et al. [5], a clade that contained the Hill partridge (*Arborophila* spp.), Crested Partridge (*Rollulus rouloul*), and Ferruginous Partridge (*Caloperdix oculea*), and the other phasianids. The second one included most of the pheasants (e.g., *Phasianus*, *Lophura*, and related genera), the typical Grey partridge and relatives (*Perdix* spp.), the turkey and the grouse. This group has been designated the “erectile clade” [17] and this study extended those findings by including the Blood Pheasant (*Ithaginis cruentus*) in the erectile clade. In addition to the strongly supported Arborophilinae and erectile clade, there was a third clade that only received marginal support. This third clade comprised junglefowl (*Gallus* spp.), peacock-pheasants (*Polyplectron* spp.), peafowl (*Pavo* spp.) and their allies within the traditional pheasants, as well as many partridges, Old World quail, and francolins (i.e., *Alectoris*, *Ammoperdix*, *Coturnix*, *Excalfactoria*, *Margaroperdix*, *Tetraogallus*, and *Francolinus*). Although there were well-supported groups within this last clade, relationships among the groups did not always receive strong support (Figure 3) and those poorly supported clades were united by short branches, consistent with the hypothesis that there was a rapid radiation.

### The Performance of Gene Tree – Species Tree Methods

**Figure 4:**
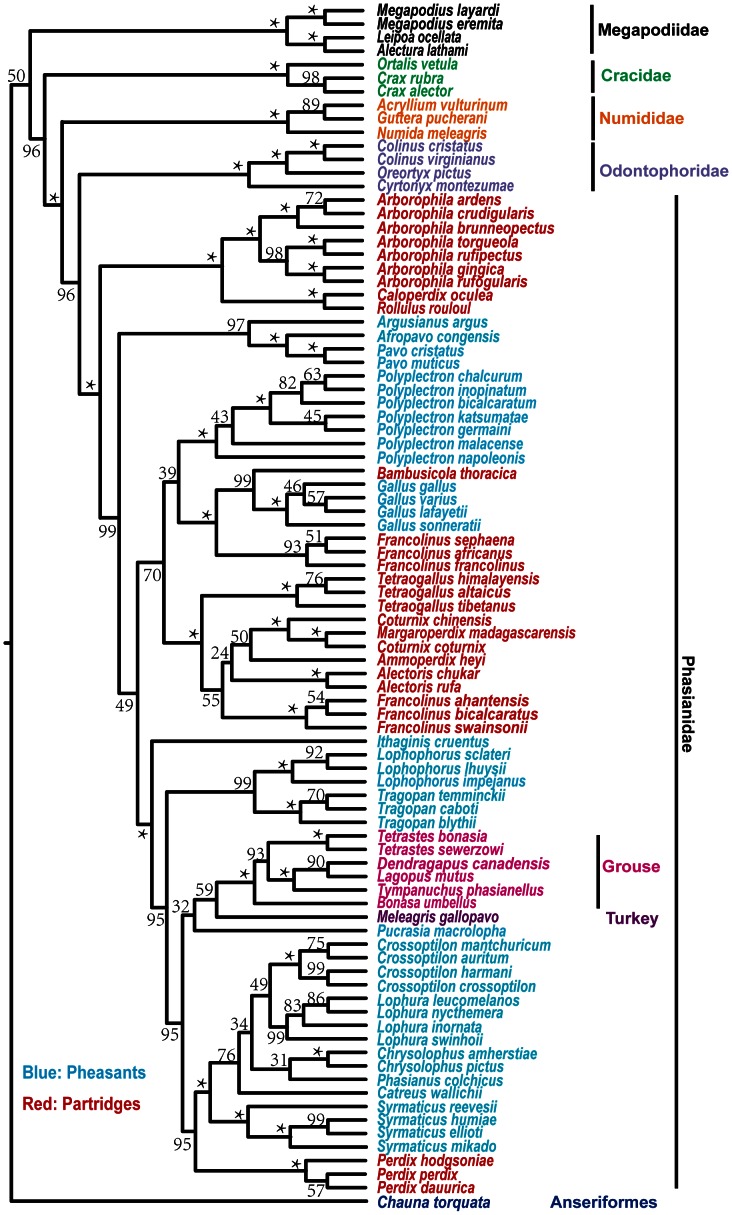
Estimate of Galliformes phylogeny obtained using NJst. Support values reflect 500 bootstrap replicates. *indicates 100% support. Other species tree methods are included in the Treefile S1. Groups of taxa are indicated using the same color coding as Figure 1.

Analyses of concatenated sequences can be an inconsistent estimator of the species tree [55] so we also used gene trees to obtain an estimate of the species tree using NJst (Figure 4) and STAR (Supporting information Treefile S1). These trees were very similar and only differed between poorly supported nodes (e.g., typically fewer than 50% of input species trees had the node in question). In terms of the basal structure of the galliforms, the NJst tree placed the peafowl clade (i.e., *Pavo*, *Afropavo*, and *Argusianus*) sister to all phasianids except the Arborophilinae (albeit with less than 50% support). In contrast, analyses of concatenated data (e.g., Figures 2 and 3) and STAR (Supporting information Treefile S1) placed the peafowl clade into a larger clade comprising junglefowls, peacock-pheasants, Old World quail, francolins, and many partridges. However, this difference appeared to reflect the exclusion of SERPINB14 from the STAR analysis; the NJst tree estimated without SERPINB14 (Supporting information Treefile S1) placed the peafowl clade in the same position as the concatenated analyses (Figures 2 and 3) and the STAR analysis. This suggested that SERPINB14 might have a strong signal that differs from the other loci included in our analysis, possibly reflecting a distinct gene tree.

There were several other differences within the major phasianid clades when comparing the gene tree-species tree analyses with the concatenated analyses. However, as with the position of the peafowl clade above, all of these relationships were poorly supported in the NJst tree and did not consistently present in the other analyses (e.g., Figure 3). For example, within the erectile clade, the position of the Cheer Pheasant (*Catreus wallichii*) differed between Figures 3 and 4. Additionally, within the third clade in the Phasianidae (which included the clades with the junglefowl, peacock-pheasants, and most partridges and francolins), relationships among the different groups varied among all of the analyses (e.g., compared Figures 2, 3, and 4). However, while there were some differences, all of the consistent and well-supported nodes in the concatenated analyses were also in the species trees estimated from gene trees, suggesting that our conclusions were robust to type of analysis.

### Tests of Tree Robustness and Potential Artifacts

Partitioned ML bootstrap values, Bayesian posterior probabilities, and species-tree (e.g., NJst) bootstrap values all provided strong support for many phylogenetic relationships (Figures 3 and 4). Gene jackknifing in which one locus was excluded at a time also resulted in tree topologies very similar to those given by the concatenated dataset (i.e. the differences among them were similar to the differences between un-partitioned and partitioned ML analyses, and these differences primarily occurred in the third phasianid clade, see Figure S4 and Treefile S1), indicating that no single gene had a strong impact on our phylogeny.

It is noteworthy that ML trees built by the Mt genes alone showed a number of differences from those obtained with the concatenated dataset (Figure 3, Figure S4 and Treefile S1) due to the reduction or even the loss of support of certain clades. For example, the third major clade within Phasianidae identified in the concatenated dataset was not found in analyses of the Mt genes. Since there were only two Mt genes included in our study, their power to resolve difficult relationships within Galliformes might be limited. Moreover, the CI and RI were greater for the nuclear than the Mt data, suggesting that the Mt data exhibited more homoplasy than the nuclear introns (Table 1). For these reasons we have more confidence in the analyses that included nuclear data than the Mt only dataset.

None of the eight partitions exhibited substitutional saturation based upon the *I*_ss_ metric [53] (Table 2). ND2 did exhibit base composition heterogeneity but excluding ND2 from the analyses had little impact on the tree topology (Figure S4 and Treefile S1). Moreover, the topology of partitioned and unpartitioned ML trees obtained after RY coding were the same for higher-level relationships as those obtained using all four nucleotides, though some lower-level relationships among genera within the Phasianidae were different (Supporting information Treefile S1). Since there were more very short branches on the RY trees, it suggested that RY-coding might lead to a loss of more informative sites and thus have a reduced power to resolve phylogenetic relationships.

**Table 2:** Test of base composition heterogeneity and substitutional saturation.

Locus	Iss	Iss.c	P	Category	χ^2^ (p)^1^
12S	0.151	0.716	0	No saturation	0.996
ND2	0.262	0.746	0	No saturation	0*
CLTC	0.18	0.684	0	No saturation	1
CLTCL1	0.184	0.683	0	No saturation	1
RHO	0.212	0.683	0	No saturation	0.984
SERPINB14	0.13	0.706	0	No saturation	1
FGB	0.173	0.727	0	No saturation	1
EEF2	0.101	0.743	0	No saturation	0.858

1 P-value for χ^2^ test of compositional homogeneity.

* indicates significant deviation.

## Discussion

There have been substantial changes over time regarding relationships among members of the Galliformes (Figure 1, Figure S1). In fact, despite the similarities evident in parts of previous large-scale trees (compare Figure 1C and 1D), a clear consensus tree regarding relationships within this order has not emerged and even the most recent studies exhibit incongruence (Figure S1). Some of this incongruence may reflect the types of data used in each analysis. For example, differences between trees based upon cladistic analyses of M/B traits [16] and those based upon molecular data could reflect the limited number of traits included in M/B data matrices. Moreover, M/B traits have the potential to be scored incorrectly [56]. Patterns of Mt sequence evolution are very complex and some analyses of Mt sequences have recovered phylogenies that are likely to reflect bias [57]. The Mt genome represents a single genetic locus so the Mt gene tree may differ from the species tree due to lineage sorting or introgression [58]. Nuclear introns show a relatively slower rate of evolution and less homoplasy than Mt sequences [15], [54], [59], but indels (insertions and deletions) in nuclear non-coding data can make alignment difficult and influence downstream evolutionary analyses [60]. We examined the influence of sequence alignment here by analyzing alternative alignments, finding that alignment had a limited influence on the tree topology (Figure S2), in agreement with other recent studies using avian introns [61], [62]. Other nuclear regions, including coding exons and untranslated regions (UTRs) typically accumulate substitutions more slowly, limiting their power to resolve rapid evolutionary radiations [63], [64]. Finally, rare genomic changes such as TE insertions accumulate very slowly and therefore have little homoplasy, but their very low rate of accumulation also limits their power [65]. Moreover, they are subject to lineage sorting, like all loci, and they do not appear to be completely homoplasy-free [66], [67]. Finally, large-scale “supermatrix” analyses that combine multiple gene regions (and M/B characters in the case of Crowe et al. [5]) have substantial missing data and the impact of this missing data upon analyses is unclear [68], [69]. Analyses using all of these markers, either independently or in various combinations, have been applied to galliform phylogeny (Figure S1) and this has clearly resulted in substantial progress toward resolving specific relationships within the order. However, a number of questions remain despite this progress; we discuss the ways that our data address these remaining questions below.

### The Deepest Branching Clades within Galliformes

Galliform phylogeny has been an active area of research for almost a century, and our understanding of the evolution of this group continues to improve with new techniques and methods. In the pre-molecular classification world, Wetmore [70] splits the Galliformes into two large groups: the superfamily Cracoidea that includes Megapodiidae and Cracidae, and the superfamily Phasianoidea that includes Tetraonidae, Phasianidae (which he defines as including New and Old World quail, pheasants, and partridges), Numididae, and Meleagrididae. Similar division suggesting a sister group between Megapodiidae and Cracidae is also found in studies that use appendicular musculature [71], [72], and DNA-DNA hybridization [18](Figure 1B). However, with the explosion of DNA sequence data there is now increasing evidence indicating that the megapodes and cracids do not form a clade. In fact, the megapodes are sister to a clade comprising cracids and the remainder of the Phasianoidea, which is further corroborated by analyses of many different types of data including extensive M/B characters [16], mitochondrial (Mt) sequences [73], combined M/B and molecular datasets [5], nuclear sequence data [13], and retrotransposon insertion [31] (Figure S1). All of our analyses revealed strong support for the hypothesis that the deepest divergence within extant Galliformes was between the megapodes and all other galliform species, with the next divergence corresponding to that between cracids and Phasianoidea.

### The Position of New World Quail and Guineafowls

The position of the New World quail has varied substantially among studies (Figure S1). Traditionally, the New World quail are considered part of the Phasianidae [70], although placement within the Phasianidae varies and includes grouping with the grouse and turkeys [74] or the Old World quail [16], [75], [76]. On the other hand, the New World quail have also been placed in their own family, Odontophoridae. Using DNA–DNA hybridization, Sibley and Ahlquist [18] place the New World quail sister to a clade comprising Numididae, Phasianidae, Meleagrididae, and Tetraonidae. This relatively deep-branching placement of New World quail is also supported by some analyses of Mt genes [14], [73], [77], though there appears to be conflict among different Mt genes [59]. The majority of nuclear introns, UTRs and Chicken Repeat 1 retroposon insertions have supported an Odontophoridae-Phasianidae-Meleagrididae-Tetraonidae clade, with Numididae sister to this group [5], [17], [31], [59], [64], [78]. This pattern was strongly supported both by concatenated (Figure 3) and species tree (Figure 4) analyses in our study, which suggested that the New World quail should be an independent family sister to the Phasianidae. However, when using the Mt genes alone, the ML bootstrap supports reduced to around 72% (Figure 3). This was consistent with the conflicting signal observed by Cox et al. [59] and might reflect the greater homoplasy exhibited by mitochondrial sequences.

### The Phylogenetic Position of Turkeys and Grouse

The turkeys and grouse have also been treated as independent families in some taxonomies [70], implying that they are distantly related to other phasianids [20]. This probably reflects the perception that these taxa have a number of special characteristics. For example, grouse are distinguished by morphological adaptations to cold environments such as feathered nostrils and tarsi. Their toes can also grow feathers or small scales in winter to adapt walking on snow and burrowing into it for shelter [70]. In contrast, both turkey species are bare headed and males have a snood (a distinctive fleshy wattle or protuberance that hangs from the top of their beak). However, more recent studies have suggested a derived position for turkeys and grouse within the Phasianidae [4], [30], [79](Figure S1A–D, F–M), even placing them sister to each other [8], [14], [80](Figure 1D, Figure S1B, D, G–L). In our study, the turkey and grouse formed a sister group nesting inside the Phasianidae. Although the support for the sister relationship varied across analyses, and sometimes was very low (less than 50%, Figures 3 and 4), the turkey-grouse clade nested into a larger clade with strong support that included taxa such as *Perdix*, *Tragopan*, and the typical pheasants (i.e., the erectile clade of Kimball and Braun [17], see discussion below). This provided strong support that the position of turkeys and grouse should be within the Phasianidae rather than considered as independent families. Since our sampling was greatest within the Phasianidae, our following discussion will be focusing on the remaining relationships within this family.

### Phylogenetic Relationships within Phasianidae

The Phasianidae has received extensive study by ornithologists, in part due to the inclusion of important avian model systems in this family, such as the chicken, quail, and turkey (see above). Since the Phasianidae includes ∼61% of galliform species, understanding relationships within the Phasianidae is critical to clarify evolutionary patterns across the Galliformes. However, previous studies have typically obtained low bootstrap support for branching order for some relationships. While failure to obtain robust support has been suggested to be due to a rapid radiation [14], [17], [81], [82], it may also be that limited marker and taxon sampling have contributed to the difficulties in resolving branching patterns and thus the different conclusions among studies.

#### “Pheasants” and “partridges”

The Phasianidae have often been split into the tribes Phasianini (pheasants) and Perdicini (partridges and allies) [18]–[21]. These tribes have typically been assumed to be closely related but reciprocally monophyletic, although there has been some debate regarding the exact sets of taxa they comprise. According to this classification, pheasants are relatively large with most species exhibiting extreme sexual dichromatism and specialized ornamental traits [4]. In contrast, partridges (including Old World quail) are often monochromatic or exhibit limited sexual dimorphism and are primarily dull colored. As these tribes have been typically defined (see above), the partridges exhibit either none or many fewer of the extreme or highly specialized ornamental traits found in the pheasants [14].

However, monophyly of the pheasants and partridges has not been supported in most recent studies. For example, there has been strong support for the sister relationship between the junglefowl (typically considered a pheasant) and the bamboo partridges (*Bambusicola* spp.) using various markers, such as mitochondrial sequences [14], [81], [83], nuclear genes [15], combined Mt and nuclear sequences [4], [17], [84], [85], combined morphological and molecular data [5] and retrotransposable elements [30], similar to what we found (Figures 2 and 4). We also identified several other examples that reject monophyly of the “pheasants” and “partridges” as traditionally defined (discussed below).

#### Clade 1: Arborophilinae

The first clade in the Phasianidae formed the earliest diverging group including three traditional partridge genera: *Arborophila* spp., *Rollulus rouloul* and *Caloperdix oculea*. This clade is also supported by other studies [4], [5](Figure 1C), though this is the first study to include nuclear sequence data from *Caloperdix* and to strongly support placement of this partridge in this clade.

#### Clade 2: “Erectile Clade”

The second major clade within Phasianidae corresponded to the “erectile clade” of Kimball and Braun [17]. In this study, we used more species but the circumscription of this group remained similar to that reported by Kimball and Braun [17] in that it contained species belonging to the traditional “Gallopheasants and allies”, “Tragopan and allies” [20], the *Perdix* partridges, as well as turkeys and grouse.

The Gallopheasant group [20] was monophyletic and it comprised six genera: *Catreus*, *Chrysolophus, Crossoptilon, Lophura*, *Phasianus* and *Syrmaticus*. Supports for many of the relationships among these taxa were low (Figures 3 and 4), though the initial divergence between *Syrmaticus* and the remaining Gallopheasants was strongly supported by all of our analyses and by a number of previous analyses (Figure 1D, Figure S1A, F, K–L, N). The sister group of the gallopheasants was *Perdix* spp. (a partridge) with 100% support (hereafter we refer to this group as the *Perdix*-Gallopheasant clade). The phylogenetic position of *Perdix* has varied among studies, being placed with *Francolinus* and other traditional partridges using morphology [16], [20], or sister to turkey and grouse in some studies based on molecular data [5], [73], [79], [81], though typically with limited support. Our analyses rejected those placements, although we noted that our analyses were in agreement with some molecular studies (Figure1D, Figure S1J–L, N). Our strong support for the *Perdix*-Gallopheasant clade further refuted the traditional division of pheasants and partridges.

Johnsgard [20] defines the “Tragopan and allies” as including *Tragopan* spp., *Lophophorus* spp., *Ithaginis cruentus*, and *Pucrasia macrolopha*. However, recent studies have not found that these taxa form a monophyletic group, in agreement with our results (Figures 2 and 4). Our data united *Tragopan* and *Lophophorus*, as have many previous studies (Figure S1), but placed *Pucrasia* and *Ithaginis* in separate positions. More specifically, our study generally placed *Pucrasia* sister to the turkey and grouse with limited support (Figures 3 and 4), though the Mt genes and the species tree analyses that lacked SERPINB14 united *Pucrasia* with *Perdix*-Gallopheasants (Supporting information Treefile S1). The results of other studies are mostly consistent with our Mt results, but with varying degrees of support. Thus, the position of *Pucrasia* cannot be established with confidence at this time. The position of *Ithaginis cruentus* has not been extensively studied. Previous studies place it at the base of the major “partridges” including the New World quail [16], or sister to Gallopheasants [5], [32], but always with marginal support. In contrast, our data strongly supported (with 100% support in all analyses, Figures 3 and 4) placing *Ithaginis* sister to the other members of the second major (or “erectile”) clade [78]. Thus, *Ithaginis* was not only distinct from the “Tragopan and allies” group of Johnsgard [20], but also distinct from the other lineages within the erectile clade.

#### Clade 3: “Chickens and allies”

In contrast to the Arborophilinae and the erectile clade that were each supported at 100% in all of our analyses, the third clade within the Phasianidae received mixed support across analyses, with analyses of the Mt genes (Figure 3) and the NJst (Figure 4) failing to support the clade. Within this group of taxa, however, were four clades each of which formed a robust monophyletic group. However, relationships among these four clades received marginal support, even with the concatenated dataset, and so relationships among the clades remained to be determined.

These four small clades included the *Gallus-Bambusicola*-*Francolinus* lineage mentioned above. Sister to this was a lineage containing *Alectoris*, *Tetraogallus*, *Coturnix*, *Ammoperdix*, *Margaroperdix*, and part of the genus *Francolinus* as traditionally circumscribed. It is clear that *Francolinus* is not a monophyletic genus, as has been found in other studies [5], [78], [86](also see Table S1). Moreover, *Coturnix* was paraphyletic based on all of our analyses since *C. coturnix* was sister to *Margaroperdix* rather than *C. chinensis* (suggesting that the alternative species name of *Excalfactoria chinensis* is more appropriate). Of the remaining two clades, one included a single genus (*Polyplectron*) and the other corresponded to the peafowl clade (*Pavo, Afropavo*, and *Argusianus*). Collectively, the peafowl clade and the peacock-pheasants have been suggested to form a monophyletic clade, the Pavoninae [5], which is identical to the peafowl and allies of Johnsgard [20]. Although a monophyletic Pavoninae has been found in some studies [6], [84], most studies have either not found resolution among the putative Pavoninae [14], [16], [30] or have not supported a monophyletic Pavoninae, instead placing *Polyplectron* distant from the peafowl clade (Figure 1D, Figure S1A, G, K–M). However, since none of these studies has robust support for relationships among these taxa, the exact relationship between *Polyplectron*, *Argusianus* and *Pavo/Afropavo* still needs further exploration.

## Conclusions

We have generated estimates of the large-scale structure of Galliformes phylogeny, using 88 ingroup species. Although many parts of this phylogeny were robust to analytical method and appeared to faithfully reflect evolutionary history, several uncertainties remained. Taken as a whole, we strongly corroborated the hypothesis that Galliformes can be split into five major families: Megapodiidae, Cracidae, Numididae, Odontophoridae, and Phasianidae. The earliest divergence was between Megapodiidae and other Galliformes, followed by the divergences of Cracidae, Numididae, and finally the sister group of Odontophoridae and Phasianidae. Moreover, the hypothesis that turkey and grouse, each of which has sometimes been considered to form an independent family, are instead part of the Phasianidae was also strongly corroborated. There were multiple examples that refuted the possibility that the traditional classification of “pheasants” or “partridges” represent monophyletic groups. Thus, we extended the suggestion [14] that these terms should be used to refer suites of similar morphological and behavioral traits because those groups are not monophyletic and therefore do not imply shared evolutionary history. While our study provided strong support for many relationships, including some that have been contentious, several uncertain nodes remained that will require additional study before there will be a well-resolved phylogeny of Galliformes.

## Supporting Information

Figure S1 **Additional prior hypotheses of Galliformes phylogeny.**
(DOC)

Figure S2 **Cluster analysis of RF distances among trees based on different alignments and partitioning strategies.**
(DOC)

Figure S3 **Comparison of the two Bayesian runs in the AWTY analyses.**
(DOC)

Figure S4 **Cluster analysis of RF distances among trees based on MAFFT alignments and the majority rule consensus tree of these trees.**
(DOC)

Table S1 **Name and taxonomy of the species examined.**
(DOC)

Table S2 **Names, location, and primer sequences of the eight regions.**
(DOC)

Table S3 **Genbank accession number for each sequence.**
(DOC)

Treefile S1 **The file of trees based on different analyses.**
(TXT)

## References

[1] del Hoyo J, Elliott A, Sargatal J (Eds.) (1994) Handbook of the Birds of the World, Vol. 2. New World Vultures to Guineafowls. Lynx Edicions, Barcelona.

[2] Brown WR, Hubbard SJ, Tickle C, Wilson SA (2003) The chicken as a model for large-scale analysis of vertebrate gene function. Nature Rev Genet 4: 87–98. 12560806 10.1038/nrg998

[3] Dimcheff DE, Krishnan M, Mindell DP (2001) Evolution and characterization of tetraonine endogenous retrovirus: a new virus related to avian sarcoma and leukosis viruses. J Virol 75: 2002–2009. 11160701 10.1128/JVI.75.4.2002-2009.2001 PMC115148

[4] Kimball RT, St.Mary CM, Braun EL (2011) A Macroevolutionary Perspective on Multiple Sexual Traits in the Phasianidae (Galliformes). Int J Evol Biol Doi:10.4061/2011/423938. 10.4061/2011/423938 PMC3119463 21716735

[5] Crowe TM, Bowie RCK, Bloomer P, Mandiwana TG, Hedderson TAJ, et al (2006) Phylogenetics, biogeography and classification of, and character evolution in, gamebirds (Aves: Galliformes): effects of character exclusion, data partitioning and missing data. Cladistics 22: 495–532. 10.1111/j.1096-0031.2006.00120.x 34892896

[6] Kimball RT, Braun EL, Ligon JD, Lucchini V, Randi E (2001) A molecular phylogeny of the peacock-pheasants (Galliformes: *Polyplectron* spp.) indicates loss and reduction of ornamental traits and display behaviours. Biol J Linn Soc 73: 187–198.

[7] Kolm N, Stein RW, Mooers AØ, Verspoor JJ, Cunningham EJA (2007) Can sexual selection drive female life histories? A comparative study on galliform birds. J Evol Biol 20: 627–638. 17305829 10.1111/j.1420-9101.2006.01248.x

[8] Nadeau NJ, Burke T, Mundy NI (2007) Evolution of an avian pigmentation gene correlates with a measure of sexual selection. Proc R Soc B 274: 1807–1813. 10.1098/rspb.2007.0174 PMC2270924 17504743

[9] Lislevand T, Figuerola J, Székely T (2009) Evolution of sexual size dimorphism in grouse and allies (Aves: Phasianidae) in relation to mating competition, fecundity demands and resource division. J Evol Biol 22: 1895–1905. 19682306 10.1111/j.1420-9101.2009.01802.x

[10] IUCN Red list of Threatened species Version 2012.1. Available: http://www.iucnredlist.org. Accessed 2013 April 17.

[11] Mooers AO, Heard SB, Chrostowski E (2005) Evolutionary heritage as a metric for conservation: in Phylogeny and Conservation (A. Purvis, T.L. Brooks and J.L. Gittleman, eds.) Oxford University Press, Oxford. 120–138.

[12] Minh BQ, Klaere S, von Haeseler A (2009) Taxon selection under split diversity. Syst Biol 58: 586–594. 20525611 10.1093/sysbio/syp058

[13] Hackett SJ, Kimball RT, Reddy S, Bowie RCK, Braun EL, et al (2008) A phylogenomic study of birds reveals their evolutionary history. Science 320: 1763–1767. 18583609 10.1126/science.1157704

[14] Kimball RT, Braun EL, Zwartjes PW, Crowe TM, Ligon JD (1999) A molecular phylogeny of the pheasants and partridges suggests that these lineages are not monophyletic. Mol Phylogenet Evol 11: 38–54. 10082609 10.1006/mpev.1998.0562

[15] Armstrong MH, Braun EL, Kimball RT (2001) Phylogenetic utility of avian ovomucoid intron G: a comparison of nuclear and mitochondrial phylogenetics in Galliformes. Auk 118: 799–804.

[16] Dyke GJ, Gulas BE, Crowe TM (2003) Suprageneric relationships of galliform birds (Aves, Galliformes): a cladistic analysis of morphological characters. Zool J Linn Soc 137: 227–244.

[17] Kimball RT, Braun EL (2008) A multigene phylogeny of Galliformes supports a single origin of erectile ability in non-feathered facial traits. J Avian Biol 39: 438–445.

[18] Sibley CG, Ahlquist JE (1990) Phylogeny and Classification of Birds: A Study in Molecular Evolution. New Haven: Yale University Press.

[19] Akishinonomiya F, Miyake T, Takada M, Ohno S, Kondo N (1995) The genetic link between the Chinese bamboo partridge (*Bambusicola thoracica*) and the chicken and junglefowls of the genus *Gallus* . Proc Natl Acad Sci USA 92: 11053–11056. 7479935 10.1073/pnas.92.24.11053 PMC40569

[20] Johnsgard PA (1986) The Pheasants of the World. New York: Oxford University Press.

[21] Johnsgard PA (1988) The Quails, Partridges, and Francolins of the World. Oxford: Oxford University Press.

[22] Beebe CW (1914) Preliminary pheasant studies. Zoologica 1: 261–285.

[23] Verheyen R (1956) Contribution a l’anatomie et a la systematique des Galliformes. Bull Inst Roy Sci Nat Belg 32: 1–24.

[24] Delacour J (1977) The pheasants of the world, 2nd edn. Hindhead, UK: World Pheasant Association and Spur Publications.

[25] Prager EM, Wilson AC, Osuga DT, Feeney RE (1976) Evolution of flightless land birds on southern continents: transferrin comparison shows monophyletic origin of ratites. J Mol Evol 8: 283–294. 978751 10.1007/BF01731001

[26] Jolles J, Schoentgen F, Jolles P, Wilson AC (1976) Amino acid sequence and immunological properties of chachalaca egg white lysozymes. J Mol Evol 8: 59–78. 940173 10.1007/BF01738883

[27] Gutierrez RJ, Zink RM, Yang SY (1983) Genic variation, systematic, and biogeographic relationships of some galliform birds. Auk 100: 33–47.

[28] Henderson JY, Moir AJ, Fothergill LA, Fothergill JE (1981) Sequences of sixteen phosphoserine peptides from ovalbumins of eight species. Eur J Biochem 114: 439–450. 6783411 10.1111/j.1432-1033.1981.tb05165.x

[29] Helm-Bychowski KM, Wilson AC (1986) Rates of nuclear DNA evolution in pheasant-like birds: Evidence from restriction maps. Proc Natl Acad Sci USA 83: 688–692. 3003745 10.1073/pnas.83.3.688 PMC322929

[30] Kaiser VB, Van Tuinen M, Ellegren H (2007) Insertion events of CR1 retrotransposable elements elucidate the phylogenetic branching order in galliform birds. Mol Biol Evol 24: 338–347. 17077154 10.1093/molbev/msl164

[31] Kriegs JO, Matzke AJM, Churakov G, Kuritzin A, Mayr G, et al (2007) Waves of genomic hitchhikers shed light on the evolution of gamebirds (Aves: Galliformes). BMC Evol Biol 7: 190. 17925025 10.1186/1471-2148-7-190 PMC2169234

[32] Eo SH, Bininda-Emonds ORP, Carroll JP (2009) Phylogenetic supertree of the fowls (Galloanserae, Aves). Zool Scripta 38: 465–481.

[33] Sorenson MD, Ast JC, Dimcheff DE, Yuri T, Mindell DP (1999) Primers for a PCR-based approach to mitochondrial genome sequencing in birds and other vertebrates. Mol Phylogenet Evol 12: 105–114. 10381314 10.1006/mpev.1998.0602

[34] Kimball RT, Braun EL, Bowie RCK, Braun MJ, Chojnowski JL, et al (2009) A set of resources to amplify nuclear regions across the avian genome. Mol Phylogenet Evol 50: 654–660. 19084073 10.1016/j.ympev.2008.11.018

[35] Tamura K, Dudley J, Nei M, Kumar S (2007) MEGA4: Molecular Evolutionary Genetics Analysis (MEGA) software version 4.0. Mol Biol Evol 24: 1596–1599. 17488738 10.1093/molbev/msm092

[36] Ogden TH, Rosenberg MS (2006) Multiple sequence alignment accuracy and phylogenetic inference. Syst Biol 55: 314–328. 16611602 10.1080/10635150500541730

[37] Smythe AB, Sanderson MJ, Nadler SA (2006) Nematode small subunit phylogeny correlates with alignment parameters. Syst Biol 55: 972–992. 17345678 10.1080/10635150601089001

[38] Katoh K, Asimenos G, Toh H (2009) Multiple alignment of DNA sequences with MAFFT. Methods Mol Biol 537: 39–64. 19378139 10.1007/978-1-59745-251-9_3

[39] Notredame C, Higgins DG, Heringa J (2000) T-Coffee: A novel method for fast and accurate multiple sequence alignment. J Mol Biol 302: 205–217. 10964570 10.1006/jmbi.2000.4042

[40] Edgar RC (2004) MUSCLE: multiple sequence alignment with high accuracy and high throughput. Nucl Acids Res 32: 1792–1797. 15034147 10.1093/nar/gkh340 PMC390337

[41] Stamatakis A (2006) RAxML-VI-HPC: maximum likelihood-based phylogenetic analyses with thousands of taxa and mixed models. Bioinformatics 22: 2688–2690. 16928733 10.1093/bioinformatics/btl446

[42] Robinson DF, Foulds LR (1981) Comparison of phylogenetic trees. Math Biosci 53: 131–147.

[43] Posada D, Crandall KA (1998) Modeltest: Testing the model of DNA substitution. Bioinformatics 14: 817–818. 9918953 10.1093/bioinformatics/14.9.817

[44] Swofford DL (2003) PAUP*. Phylogenetic Analysis Using Parsimony (*and Other Methods), Version 4. Sinauer Associates, Sunderland, MA.

[45] Felsenstein J (1985) Confidence limits on phylogenies: an approach using the bootstrap. Evolution 39: 783–791. 28561359 10.1111/j.1558-5646.1985.tb00420.x

[46] Ronquist F, Huelsenbeck JP (2003) MrBayes 3: Bayesian phylogenetic inference under mixed models. Bioinformatics 19: 1572–1574. 12912839 10.1093/bioinformatics/btg180

[47] Gelman A, Rubin D (1992) Inference from iterative simulation using multiple sequences. Stat Sci 7: 457–511.

[48] Rambaut A, Drummond AJ (2007) Tracer v1.4, Available: http://beast.bio.ed.ac.uk/Tracer. Accessed 2013 April 17.

[49] Wilgenbusch JC, Warren DL, Swofford DL (2004) AWTY: A system for graphical exploration of MCMC convergence in Bayesian phylogenetic inference. Available: http://ceb.csit.fsu.edu/awty. Accessed 2013 April 17. 10.1093/bioinformatics/btm388 17766271

[50] Liu L, Yu L (2011) Estimating species trees from unrooted gene trees. Syst Biol 60: 661–667. 21447481 10.1093/sysbio/syr027

[51] Liu L, Yu L, Pearl DK, Edwards SV (2009) Estimating species phylogenies using coalescence times among sequences. Syst Biol 58: 468–477. 20525601 10.1093/sysbio/syp031

[52] Shaw TI, Ruan Z, Glenn T, Liu L (2012) Webserver for Species Tree Reconstruction.

[53] Xia X, Xie Z, Salemi M, Chen L, Wang Y (2003) An index of substitution saturation and its application. Mol Phylogenet Evol 26: 1–7. 12470932 10.1016/s1055-7903(02)00326-3

[54] Prychitko TM, Moore WS (2000) Comparative evolution of the mitochondrial cytochrome b gene and nuclear beta-fibrinogen intron 7 in woodpeckers. Mol Biol Evol 17: 1101–1111. 10889223 10.1093/oxfordjournals.molbev.a026391

[55] Kubatko LS, Degnan JH (2007) Inconsistency of phylogenetic estimates from concatenated data under coalescence. Syst Biol 56: 17–24. 17366134 10.1080/10635150601146041

[56] Mayr G (2008) The fossil record of galliform birds: comments on Crowe et al. (2006). Cladistics 24: 74–76.

[57] Braun EL, Kimball RT (2002) Examining basal avian divergences with mitochondrial sequences: model complexity, taxon sampling, and sequence length. Syst Biol 51: 614–625. 12228003 10.1080/10635150290102294

[58] McGuire JA, Linkem CW, Koo MS, Hutchison DW, Lappin AK, et al (2007) Mitochondrial introgression and incomplete lineage sorting through space and time: phylogenetics of crotaphytid lizards. Evolution 61: 2879–2897. 17941840 10.1111/j.1558-5646.2007.00239.x

[59] Cox WA, Kimball RT, Braun EL (2007) Phylogenetic position of the New World quail (Odontophoridae): Eight nuclear loci and three mitochondrial regions contradict morphology and the Sibley–Ahlquist tapestry. Auk 124: 71–84.

[60] Kruger D, Kapturska D, Fischer C, Daniel R, Wubet T (2012) Diversity measures in environmental sequences are hightly dependent on alignment quality-data from ITS and new LSU primers targeing Basidiomycetes. PLoS One. 7: e32139. 10.1371/journal.pone.0032139 PMC3283731 22363808

[61] Wang N, Braun EL, Kimball RT (2012) Testing hypotheses about the sister group of the Passeriformes using an Independent 30-locus data set. Mol Biol Evol 29: 737–750. 21940640 10.1093/molbev/msr230

[62] Smith JV, Braun El, Kimball RT (2013) Ratite non-monophyly: Independent evidence from 40 novel loci. Syst Biol 62: 35–49. 22831877 10.1093/sysbio/sys067

[63] Chojnowski JL, Kimball RT, Braun EL (2008) Introns outperform exons in analyses of basal avian phylogeny using clathrin heavy chain genes. Gene 410: 89–96. 18191344 10.1016/j.gene.2007.11.016

[64] Bonilla AJ, Braun EL, Kimball RT (2010) Comparative molecular evolution and phylogenetic utility of 3′-UTRs and introns in Galliformes. Mol Phylogenet Evol 56: 536–542. 20398778 10.1016/j.ympev.2010.04.006

[65] Braun EL, Kimball RT, Han KL, Iuhasz-Velez NR, Bonilla AJ, et al (2011) Homoplastic microinversions and the avian tree of life. BMC Evol Biol 11: 141–151. 21612607 10.1186/1471-2148-11-141 PMC3123225

[66] Han KL, Braun EL, Kimball RT, Reddy S, Bowie RCK, et al (2011) Are transposable element insertions homoplasy free?: An examination using the avian tree of life. Syst Biol 60: 375–86. 21303823 10.1093/sysbio/syq100

[67] Liu Z, He L, Yuan H, Yue B, Li J (2012) CR1 retroposons provide a new insight into the phylogeny of Phasianidae species (Aves: Galliformes). Gene 502: 125–132. 22565186 10.1016/j.gene.2012.04.068

[68] Lemmon AR, Brown JM, Stanger-Hall K, Lemmon EM (2009) The effect of ambiguous data on phylogenetic estimates obtained by maximum likelihood and Bayesian inference. Syst Biol 58: 130–145. 20525573 10.1093/sysbio/syp017 PMC7539334

[69] Roure B, Baurain D, Philippe H (2012) Impact of missing data on phylogenies inferred from empirical phylogenomic data sets. Mol Biol Evol 30: 197–214. 22930702 10.1093/molbev/mss208

[70] Wetmore A (1960) A classification for the birds of the world. Smithson Miscellaneous Collection 139: 1–37.

[71] Hudson GE, Lanzillotti PJ (1964) Muscles of the pectroal limb in galliform birds. Amer Midl Natur 71: 1–113.

[72] Hudson GE, Parker RA, Berge JV, Lanzillotti PJ (1966) A numerical analysis of the modifications of the appendicular muscles in various genera of gallinaceous birds. Amer Midl Natur 76: 1–73.

[73] Pereira SL, Baker AJ (2006) A molecular timescale for galliform birds accounting for uncertainty in time estimates and heterogeneity of rates of DNA substitutions across lineages and sites. Mol Phylogenet Evol 38: 499–509. 16112881 10.1016/j.ympev.2005.07.007

[74] Stock AD, Bunch TD (1982) The evolutionary implications of chromosome banding pattern homologies in the bird order Galliformes. Cytogenet Cell Genet 34: 136–148. 7151485 10.1159/000131802

[75] Crowe TM (1988) Molecules vs. morphology in phylogenetics: A non-controversy. Trans R Soc South Afr 46: 317–334.

[76] Randi E, Fusco G, Lorenzini R, Timothy MC (1991) Phylogenetic relationships and rates of allozyme evolution with the Phasianidae. Biochem Syst Ecol 19: 213–221.

[77] Kornegay JR, Kocher TD, Williams LA, Wilson AC (1993) Pathways of lysozyme evolution inferred from the sequences of cytochrome b in birds. J Mol Evol 37: 367–379. 8308906 10.1007/BF00178867

[78] Cohen C, Wakeling JL, Mandiwana-Neudani TG, Sande E, Dranzoa C, et al (2012) Phylogenetic affinities of evolutionary enigmatic African galliforms: the Stone Partridge *Ptilopachus petrosus* and Nahan’s Francolin *Francolinus nahani*, and support for their sister relationship within New World quails. Ibis 154: 768–780.

[79] Lucchini V, Randi E (1999) Molecular evolution of the mtDNA control-region in galliform birds. In: Adams, N.J., Slotow, R.H. (Eds.), Proceedings of the 22 International Ornithological Congress. Durban. Birdlife South Africa, Johannesburg, pp. 732–739.

[80] Shen YY, Liang L, Sun YB, Yue BS, Yang XJ, et al (2010) A mitogenomic perspective on the ancient, rapid radiation in the Galliformes with an emphasis on the Phasianidae. BMC Evol Biol 10: 132. 20444289 10.1186/1471-2148-10-132 PMC2880301

[81] Dimcheff DE, Drovetski SV, Mindell DP (2002) Phylogeny of Tetraoninae and other galliform birds using mitochondrial 12S and ND2 genes. Mol Phylogenet Evol 24: 203–215. 12144757 10.1016/s1055-7903(02)00230-0

[82] Smith EJ, Shi L, Tu Z (2005) Gallus gallus agrecan gene-based phylogenetic analysis of selected avian groups. Genetica 124: 23–32. 16011000 10.1007/s10709-004-5184-4

[83] Kan XZ, Yang JK, Li XF, Chen L, Lei ZP, et al (2010) Phylogeny of major lineages of galliform birds (Aves: Galliformes) based on complete mitochondrial genomes. Genet Mol Res 9: 1625–1633. 20730714 10.4238/vol9-3gmr898

[84] Meng Y, Dai B, Ran JH, Li J, Yue BS (2008) Phylogenetic position of the genus *Tetraophasis* (Aves, Galliformes, Phasianidae) as inferred from mitochondrial and nuclear sequences. Biochem Syst Ecol 36: 626–637.

[85] Bao XK, Liu NF, Qu JY, Wang XL, An B, et al (2010) The phylogenetic position and speciation dynamics of the genus *Perdix* (Phasianidae, Galliformes). Mol Phylogenet Evol 56: 840–847. 20363341 10.1016/j.ympev.2010.03.038

[86] Bloomer P, Crowe TM (1998) Francolin phylogenetics: molecular, morphobehavioral, and combined evidence. Mol Phylogenet Evol 9: 236–254. 9562983 10.1006/mpev.1997.0469

