# Supplementary figures and images for: Assessing Phylogenetic Relationships among Galliformes: A Multigene Phylogeny with Expanded Taxon Sampling in Phasianidae
Source: PLoS One. 2013 May 31;8(5):e64312. doi: 10.1371/journal.pone.0064312 (PMC3669371; doi:10.1371/journal.pone.0064312)

**
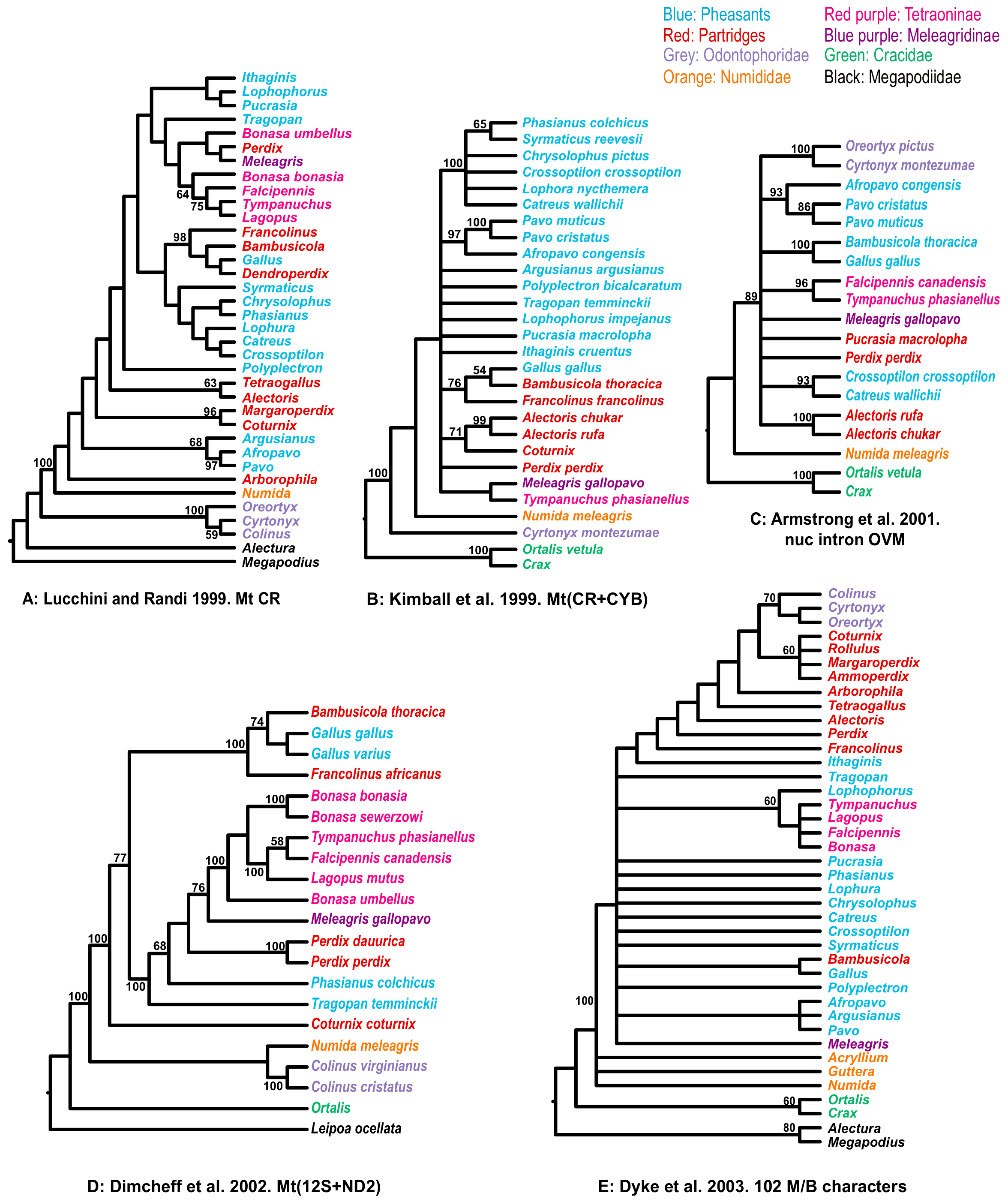
**

**
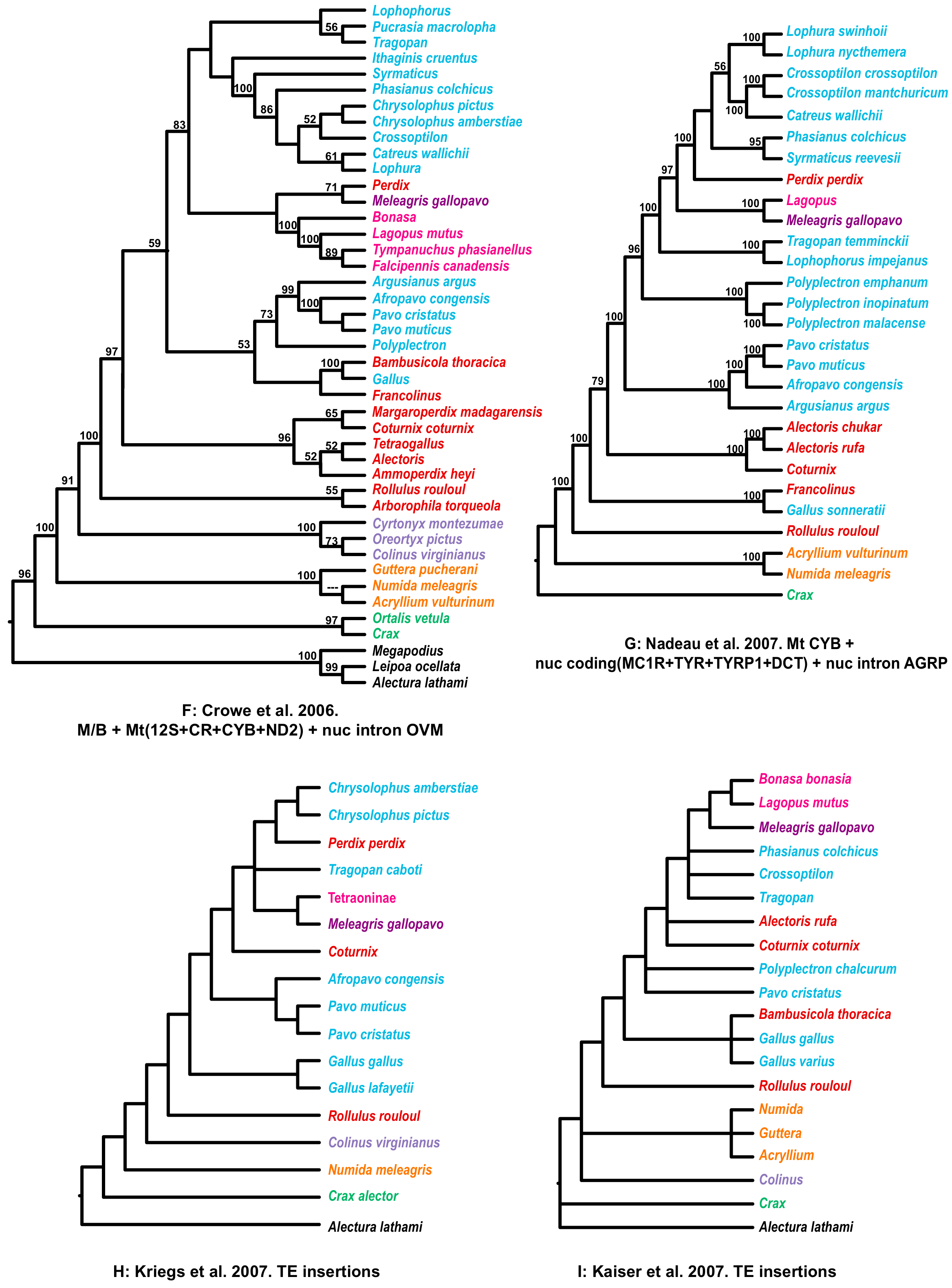
**

**
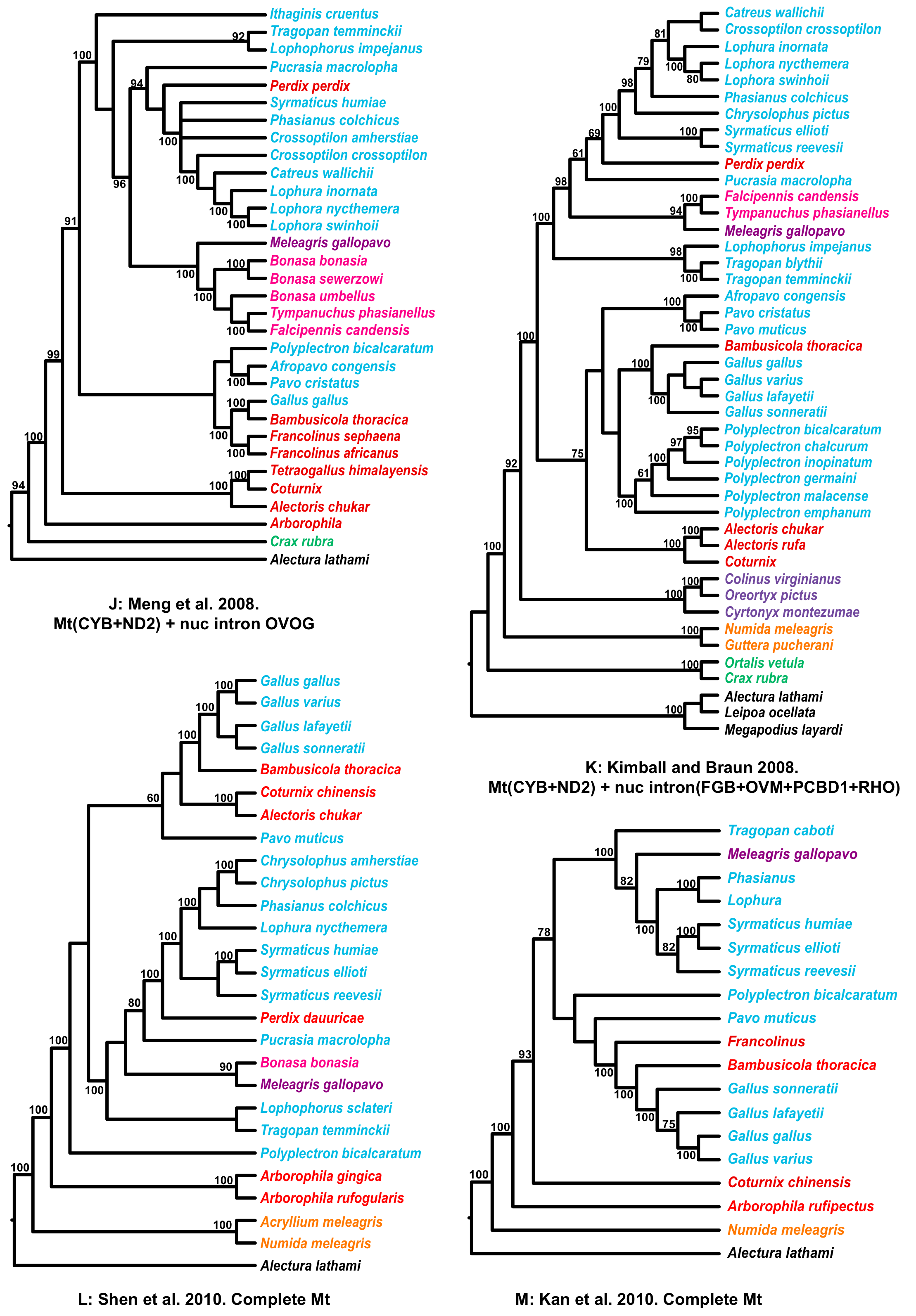
**

**
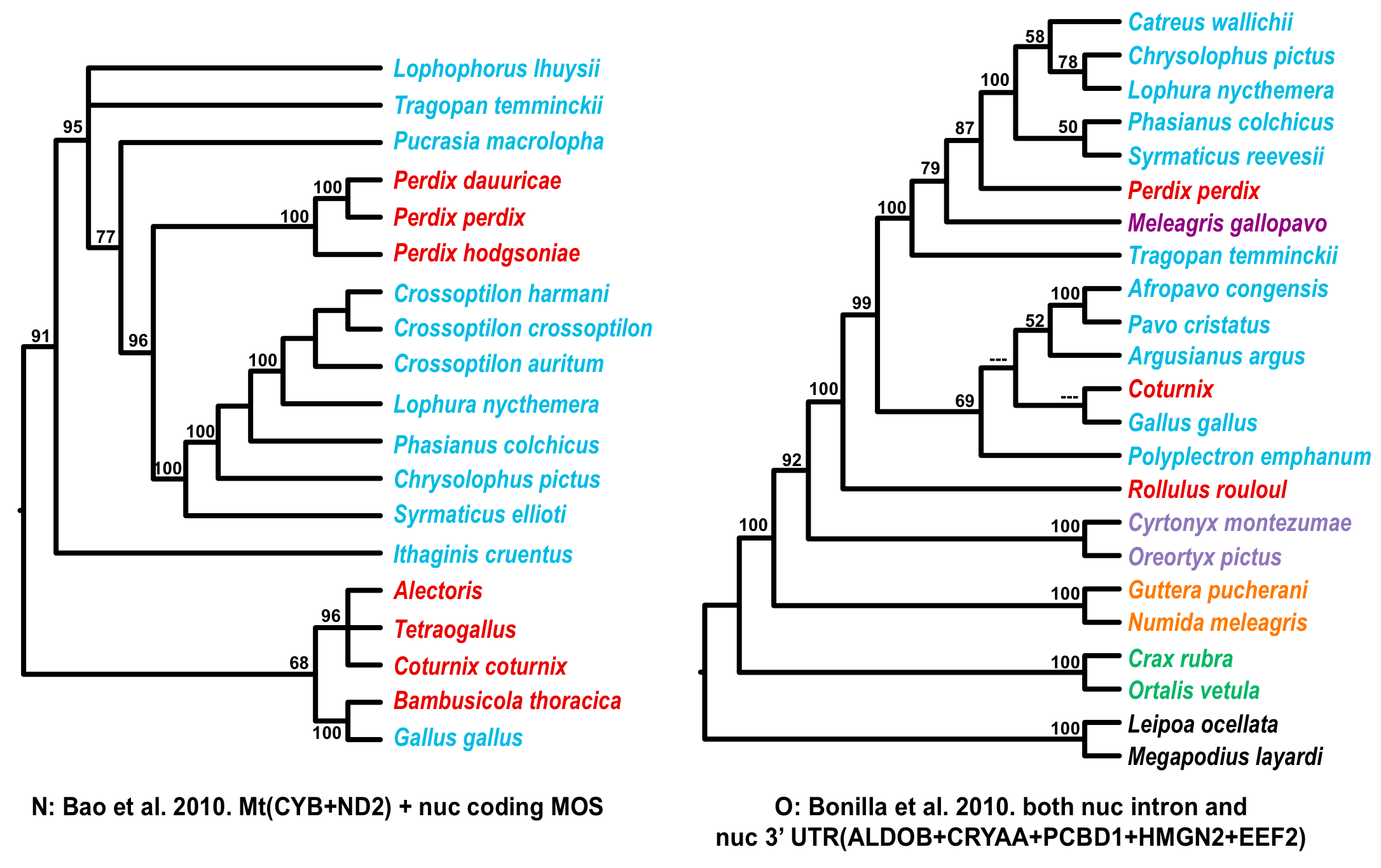
**

**Figure S1. Additional prior hypotheses of Galliformes phylogeny.**

Supplement: Figure S1 — Additional prior hypotheses of Galliformes phylogeny. (DOC) [file pone.0064312.s001.doc]
