# Supplementary material for: Assessing Phylogenetic Relationships among Galliformes: A Multigene Phylogeny with Expanded Taxon Sampling in Phasianidae
Source: PLoS One. 2013 May 31;8(5):e64312. doi: 10.1371/journal.pone.0064312 (PMC3669371; doi:10.1371/journal.pone.0064312)
Supplement: Figure S2 — Cluster analysis of RF distances among trees based on different alignments and partitioning strategies. (DOC) [file pone.0064312.s002.doc]

**Figure S2. Cluster analysis of RF distances among trees based on different alignments and partitioning strategies.** Mt: two mitochondrial DNA regions; Nuclear: six nuclear loci; all: a concatenated dataset including all sequences.
