# Supplementary material for: Assessing Phylogenetic Relationships among Galliformes: A Multigene Phylogeny with Expanded Taxon Sampling in Phasianidae
Source: PLoS One. 2013 May 31;8(5):e64312. doi: 10.1371/journal.pone.0064312 (PMC3669371; doi:10.1371/journal.pone.0064312)
Supplement: Figure S3 — Comparison of the two Bayesian runs in the AWTY analyses. (DOC) [file pone.0064312.s003.doc]

[
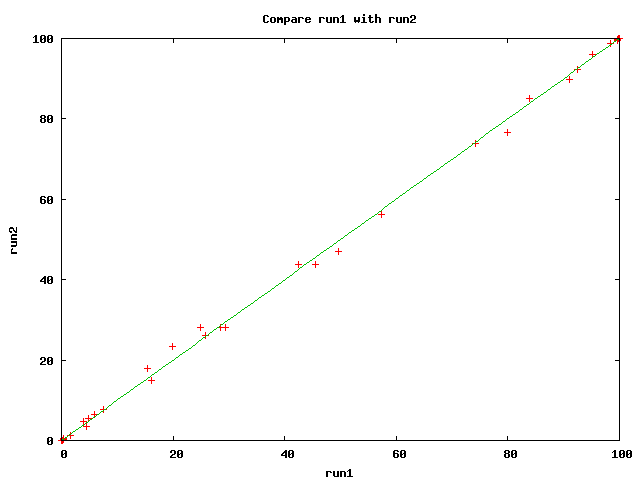
](http://king2.scs.fsu.edu/CEBProjects/awty/tmp27af0/Compare/plotYr2JSa1vs2.png)

**Figure S3. Comparison of the two Bayesian runs in the AWTY analyses.** The high correlation suggests convergence in our analyses.
