# Supplementary material for: Assessing Phylogenetic Relationships among Galliformes: A Multigene Phylogeny with Expanded Taxon Sampling in Phasianidae
Source: PLoS One. 2013 May 31;8(5):e64312. doi: 10.1371/journal.pone.0064312 (PMC3669371; doi:10.1371/journal.pone.0064312)
Supplement: Figure S4 — Cluster analysis of RF distances among trees based on MAFFT alignments and the majority rule consensus tree of these trees. (DOC) [file pone.0064312.s004.doc]

**Figure S4_A. Cluster analysis of RF distances among trees based on MAFFT alignments.** Trees based on RY-coded data, single-gene jackknifing, and two different partitioning strategies are shown**.**


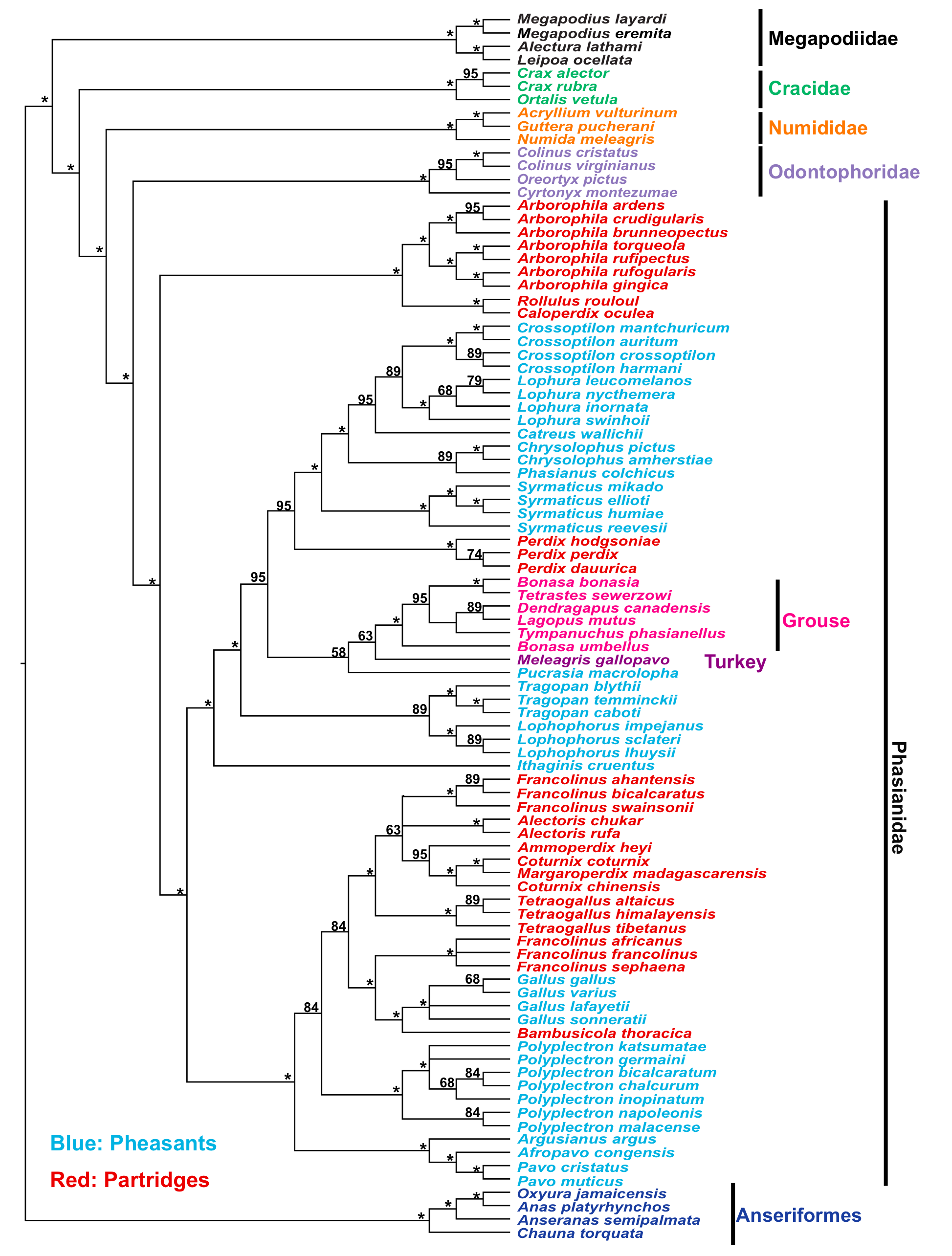


**Figure S4_B. The majority rule consensus tree of the above 19 trees in Figure S4_A.**
