# Supplementary material for: Assessing Phylogenetic Relationships among Galliformes: A Multigene Phylogeny with Expanded Taxon Sampling in Phasianidae
Source: PLoS One. 2013 May 31;8(5):e64312. doi: 10.1371/journal.pone.0064312 (PMC3669371; doi:10.1371/journal.pone.0064312)
Supplement: Table S1 — Name and taxonomy of the species examined. (DOC) [file pone.0064312.s005.doc]

**Table S1.** Name and taxonomy of the species examined.

| Family | Group | Species | Common name |
| --- | --- | --- | --- |
| Megapodiidae | Megapodes | *Alectura lathami* | Australian Brush Turkey |
|  |  | *Leipoa ocellata* | Malleefowl |
|  |  | *Megapodius eremita* | Melanesian Scrubfowl |
|  |  | *Megapodius layardi* | Vanuatu Scrubfowl |
| Cracidae | Chachalacas and Currasows | *Crax alector* | Black Curassow |
|  |  | *Crax rubra* | Great Curassow |
|  |  | *Ortalis vetula* | Plain Chachalaca |
| Numididae | Guineafowls | *Acryllium vulturinum* | Vulturine Guineafowl |
|  |  | *Guttera pucherani* | Crested Guineafowl |
|  |  | *Numida meleagris* | Helmeted Guineafowl |
| Odontophoridae | New World quail | *Colinus cristatus* | Crested Bobwhite |
|  |  | *Colinus virginianus* | Northern Bobwhite |
|  |  | *Cyrtonyx montezumae* | Montezuma Quail |
|  |  | *Oreortyx pictus* | Mountain Quail |
| Phasianidae | Turkey (Meleagrididae) | *Meleagris gallopavo* | Wild Turkey |
|  | Grouse (Tetraonidae) | *Bonasa umbellus* | Ruffed Grouse |
|  |  | *Dendragapus canadensis* | Spruce Grouse |
|  |  | *Lagopus mutus* | Rock Ptarmigan |
|  |  | *Tetrastes bonasia* | Hazel Grouse |
|  |  | *Tetrastes sewerzowi* | Chinese Grouse |
|  |  | *Tympanuchus phasianellus* | Sharp-tailed Grouse |
|  | Partridges | *Alectoris chukar* | Chukar |
|  |  | *Alectoris rufa* | Red-legged Partridge |
|  |  | *Ammoperdix heyi* | Sand Partridge |
|  |  | *Arborophila torqueola* | Hill Partridge |
|  |  | *Arborophila ardens* | Hainan Partridge |
|  |  | *Arborophila brunneopectus* | Bar-backed Partridge |
|  |  | *Arborophila crudigularis* | Taiwan Partridge |
|  |  | *Arborophila gingica* | White-necklaced Partridge |
|  |  | *Arborophila rufipectus* | Sichuan Partridge |
|  |  | *Arborophila rufogularis* | Rufous-throated Partridge |
|  |  | *Bambusicola thoracica* | Chinese Bamboo Partridge |
|  |  | *Caloperdix oculea* | Ferruginous Partridge |
|  |  | *Coturnix coturnix* | Common Quail |
|  |  | *Dendroperdix sephaena ** | Crested Francolin |
|  |  | *Excalfactoria chinensis ** | Blue-breasted Quail |
|  |  | *Francolinus francolinus* | Black Francolin |
|  |  | *Margaroperdix madagascarensis* | Madagascar Partridge |
|  |  | *Perdix dauurica* | Daurian Partridge |
|  |  | *Perdix hodgsoniae* | Tibetan Partridge |
|  |  | *Perdix perdix* | Gray Partridge |
|  |  | *Pternistis ahantensis ** | Ahanta Francolin |
|  |  | *Pternistis bicalcaratus ** | Double-spurred Francolin |
|  |  | *Pternistis swainsonii ** | Swainson's Francolin |
|  |  | *Rollulus rouloul* | Crested Partridge |
|  |  | *Scleroptila africanus ** | Gray-winged Francolin |
|  |  | *Tetraogallus altaicus* | Altai Snowcock |
|  |  | *Tetraogallus himalayensis* | Himalayan Snowcock |
|  |  | *Tetraogallus tibetanus* | Tibetan Snowcock |
|  | Pheasants | *Afropavo congensis* | Congo Peacock |
|  |  | *Argusianus argus* | Great Argus |
|  |  | *Catreus wallichii* | Cheer Pheasant |
|  |  | *Chrysolophus amherstiae* | Lady Amherst's Pheasant |
|  |  | *Chrysolophus pictus* | Golden Pheasant |
|  |  | *Crossoptilon auritum* | Blue Eared Pheasant |
|  |  | *Crossoptilon crossoptilon* | White Eared Pheasant |
|  |  | *Crossoptilon harmani* | Tibetan Eared Pheasant |
|  |  | *Crossoptilon mantchuricum* | Brown Eared Pheasant |
|  |  | *Gallus gallus* | Red Junglefowl |
|  |  | *Gallus lafayetii* | Ceylon Junglefowl |
|  |  | *Gallus sonneratii* | Gray Junglefowl |
|  |  | *Gallus varius* | Green Junglefowl |
|  |  | *Ithaginis cruentus* | Blood Pheasant |
|  |  | *Lophophorus impejanus* | Himalayan Monal |
|  |  | *Lophophorus lhuysii* | Chinese Monal |
|  |  | *Lophophorus sclateri* | Sclater's Monal |
|  |  | *Lophura inornata* | Salvadori's Pheasant |
|  |  | *Lophura leucomelanos* | Kalij Pheasant |
|  |  | *Lophura nycthemera* | Silver Pheasant |
|  |  | *Lophura swinhoii* | Swinhoe's Pheasant |
|  |  | *Pavo cristatus* | Indian Peafowl |
|  |  | *Pavo muticus* | Green Peafowl |
|  |  | *Phasianus colchicus* | Ring-necked Pheasant |
|  |  | *Polyplectron bicalcaratum* | Gray Peacock Pheasant |
|  |  | *Polyplectron chalcurum* | Bronze-tailed Peacock Pheasant |
|  |  | *Polyplectron germaini* | Germain's Peacock Pheasant |
|  |  | *Polyplectron inopinatum* | Mountain Peacock Pheasant |
|  |  | *Polyplectron katsumatae* | Hainan Peacock Pheasant |
|  |  | *Polyplectron malacense* | Malayan Peacock Pheasant |
|  |  | *Polyplectron napoleonis* | Palawan Peacock Pheasant |
|  |  | *Pucrasia macrolopha* | Koklass Pheasant |
|  |  | *Syrmaticus ellioti* | Elliot's Pheasant |
|  |  | *Syrmaticus humiae* | Hume's Pheasant |
|  |  | *Syrmaticus mikado* | Mikado Pheasant |
|  |  | *Syrmaticus reevesii* | Reeves's Pheasant |
|  |  | *Tragopan blythii* | Blyth's Tragopan |
|  |  | *Tragopan caboti* | Cabot's Tragopan |
|  |  | *Tragopan temminckii* | Temminck's Tragopan |
| Anseriformes | (outgroups) | *Anas platyrhynchos* | Common Mallard |
|  |  | *Anseranas semipalmata* | Magpie Goose |
|  |  | *Chauna torquata* | Southern Screamer |
|  |  | *Oxyura jamaicensis* | Ruddy Duck |

*** *Dendroperdix*, *Pternistes*, and *Scleroptila* are often placed in the genus *Francolinus*, but are not monophyletic (see Crowe et al. 2006); *Excalfactoria* is often referred to as *Coturnix*.
