# Supplementary material for: Assessing Phylogenetic Relationships among Galliformes: A Multigene Phylogeny with Expanded Taxon Sampling in Phasianidae
Source: PLoS One. 2013 May 31;8(5):e64312. doi: 10.1371/journal.pone.0064312 (PMC3669371; doi:10.1371/journal.pone.0064312)
Supplement: Table S2 — Names, location, and primer sequences of the eight regions. (DOC) [file pone.0064312.s006.doc]

**Table S2.** Names, location, and primer sequences of the eight regions.

| Location | HUGO name and description | Primers (5'-3') |
| --- | --- | --- |
| Nuclear^a^ | **CLTC** (intron7) | CAGAATCCTGATCTAGCTTTACGAATGGC |
|  | Clathrin heavy polypeptide | CATTTCTCCAGAAGTTGTTTGCGTCC |
|  | **CLTCL1** (intron 7) | CACCAATGTTCTGCAGAATCCTGA |
|  | Clathrin heavy chain-like 1 | CCAGCTTATCTTCCTTNAGCCATTTCTC |
|  | **EEF2** (introns 5 and 6) | GAAACAGTTTGCTGAGATGTATGTTGC |
|  | Eukaryotic Translation Elongation factor 2 | GGTTTGCCCTCCTTGTCCTTATC |
|  | **FGB** (intron 5) | CACGCCATATAGAGTATACTGTGACA |
|  | Beta-fibrinogen | AACACTACCATCCTGGCGATTCTGAA |
|  | **Rho** (intron 1) | GAACGGGTACTTTGTCTTTGGAGTAAC |
|  | Rhodopsin | CCCATGATGGCGTGGTTCTCCCC |
|  | **Serpinb14** (intron C)* | GTTCGCTTTGATAAACTTCCAGG |
|  | Ovalbumin | GGTGATTTGGTTGAGNATGTC |
| Mitochondrial^b^ | **MT-RNR1 (12S)** | ACACAAAGCATGGCACTGAA |
|  | 12S ribosomal RNA | CTTTCAGGTGTAAGCTGARTGCTT |
|  | **MT-ND2** | GCCCATACCCCRAAAATG |
|  | NADH dehydrogenase 2 | CCTTATTTAAGGCTTTGAAGGC |

^a^: nuclear primers are present in Kimball et al. (2009); *: unpublished primers

^b^: nuclear primers are present in Sorenson et al. (1999)
