# Supplementary material for: Assessing Phylogenetic Relationships among Galliformes: A Multigene Phylogeny with Expanded Taxon Sampling in Phasianidae
Source: PLoS One. 2013 May 31;8(5):e64312. doi: 10.1371/journal.pone.0064312 (PMC3669371; doi:10.1371/journal.pone.0064312)
Supplement: Table S3 — Genbank accession number for each sequence. (DOC) [file pone.0064312.s007.doc]

**Table S3.** Genbank accession number for each sequence.

| **Species** | **MT-12S** | **MT-ND2** | **CLTC** | **CLTCL1** | **EEF2** | **FGB** | **RHO** | **SERPINB14** |
| --- | --- | --- | --- | --- | --- | --- | --- | --- |
| *Alectura lathami* | AY274004 | AF394616 | KC749575 | KC749620 | KC749687 | KC749717 | ----------- | KC749863 |
| *Acryllium vulturinum* | KC785619 | KC785697 | KC785633 | KC785647 | KC785664 | KC785681 | KC785714 | KC785731 |
| *Afropavo congensis* | KC749446 | DQ307000 | KC749573 | KC749618 | FJ881857 | KC749715 | EF569434 | KC749861 |
| *Alectoris chukar* | KC749447 | DQ307001 | KC749574 | KC749619 | KC749686 | KC749716 | EF569435 | KC749862 |
| *Alectoris rufa* | KC749448 | DQ307002 | KC749576 | KC749621 | KC749688 | KC749718 | EF569436 | KC749864 |
| *Ammoperdix heyi* | KC785603 | KC785682 | KC785620 | KC785634 | KC785648 | KC785665 | KC785698 | KC785715 |
| *Anas platyrhynchos* | EU009397 | AF059142 | EU738108 | EU805796 | EU738565 | EU739196 | EU737158 | ----------- |
| *Anseranas semipalmata* | DQ674553 | AY274054 | EU738115 | JF496924 | EU738578 | EU739210 | EU737172 | ----------- |
| *Arborophila ardens* | KC778960 | KC778809 | KC778939 | KC778831 | KC778853 | KC778918 | KC778897 | KC778875 |
| *Arborophila brunneopectus* | KC778961 | KC778810 | KC778940 | KC778834 | KC778854 | KC778919 | KC778898 | KC778876 |
| *Arborophila crudigularis* | KC778962 | KC778811 | KC778941 | KC778832 | KC778855 | KC778920 | KC778899 | KC778877 |
| *Arborophila gingica* | KC778963 | KC778812 | KC778942 | KC778833 | KC778856 | KC778921 | KC778900 | KC778878 |
| *Arborophila rufipectus* | KC778975 | KC778824 | KC778954 | KC778846 | KC778868 | ----------- | KC778912 | KC778890 |
| *Arborophila rufogularis* | KC778976 | KC778825 | KC778955 | KC778847 | KC778869 | KC778933 | KC778913 | KC778891 |
| *Arborophila torqueola* | KC785604 | KC785683 | KC785621 | KC785635 | KC785649 | KC785666 | KC785700 | KC785716 |
| *Argusianus argus* | KC749449 | KC749858 | KC749577 | KC749622 | KC749689 | KC749719 | KC785701 | KC749865 |
| *Bambusicola thoracica* | KC749450 | AF222538 | KC749578 | KC749623 | KC749690 | KC749720 | EF569437 | KC749866 |
| *Bonasa umbellus* | KC785605 | KC785684 | KC785622 | KC785636 | KC785650 | KC785667 | KC785700 | KC785717 |
| *Caloperdix oculea* | KC785606 | KC785685 | KC785623 | KC785637 | KC785651 | KC785668 | KC785701 | KC785718 |
| *Catreus wallichii* | KC749451 | DQ307003 | KC749579 | KC749624 | FJ881849 | KC749721 | EF569438 | KC749867 |
| *Chauna torquata* | AY274006 | AY274053 | EU738133 | JF496940 | EU738598 | EU739229 | EU737192 | ----------- |
| *Chrysolophus amherstiae* | KC778965 | KC778814 | KC778944 | KC778836 | KC778858 | KC778923 | KC778902 | KC778880 |
| *Chrysolophus pictus* | KC749452 | DQ307004 | KC749580 | KC749625 | FJ881850 | KC749722 | EF569439 | KC749868 |
| *Colinus cristatus* | AF222575 | ----------- | EU738137 | JF496944 | EU738604 | EU739234 | EU737198 | ----------- |
| *Colinus virginianus* | AF222576 | AF222545 | KC749582 | KC749627 | KC749692 | KC749724 | AY952753 | KC749870 |
| *Coturnix coturnix* | AP003195 | AP003195 | KC749581 | KC749626 | KC749691 | KC749723 | AY952756 | KC749869 |
| *Crax alector* | ----------- | AY141931 | EU302719 | EU302762 | EU738611 | EU739241 | EU737204 | ----------- |
| *Crax rubra* | AY274003 | AY952746 | KC749583 | KC749628 | FJ881845 | KC749725 | AY952750 | KC749871 |
| *Crossoptilon auritum* | KC778966 | KC778815 | KC778945 | KC778837 | KC778859 | KC778924 | KC778903 | KC778881 |
| *Crossoptilon crossoptilon* | KC749453 | DQ307005 | KC749584 | KC749629 | KC749693 | KC749726 | EF569440 | KC749872 |
| *Crossoptilon harmani* | KC778967 | KC778816 | KC778946 | KC778838 | KC778860 | KC778925 | KC778904 | KC778882 |
| *Crossoptilon mantchuricum* | KC778968 | KC778817 | KC778947 | KC778839 | KC778861 | KC778926 | KC778905 | KC778883 |
| *Cyrtonyx montezumae* | AY952764 | AY952748 | KC749585 | KC749630 | FJ881848 | KC749727 | AY952754 | KC749873 |
| *Dendragapus canadensis* | KC749454 | DQ307006 | KC749586 | KC749631 | KC749694 | KC749728 | EF569441 | KC749874 |
| *Dendroperdix sephaena ** | KC785612 | KC785690 | ----------- | ----------- | KC785657 | KC785674 | KC785707 | KC785724 |
| *Excalfactoria chinensis ** | KC785607 | KC785686 | KC785624 | KC785638 | KC785652 | KC785669 | KC785702 | KC785719 |
| *Francolinus francolinus* | KC785611 | ----------- | KC785627 | KC785642 | KC785656 | KC785673 | KC785706 | KC785723 |
| *Gallus gallus* | X52392 | X52392 | KC749587 | KC749632 | FJ881855 | KC749729 | AY952757 | KC749875 |
| *Gallus lafayetii* | KC749455 | AP003325 | KC749588 | KC749633 | KC749695 | KC749730 | EF569442 | KC749876 |
| *Gallus sonneratii* | KC749456 | AP006746 | KC749589 | KC749634 | KC749696 | KC749731 | EF569443 | KC749877 |
| *Gallus varius* | KC749457 | AF222551 | KC749591 | KC749636 | KC749697 | KC749733 | EF569444 | KC749879 |
| *Guttera pucherani* | AY952763 | AY952747 | KC749590 | KC749635 | FJ881847 | KC749732 | AY952752 | KC749878 |
| *Ithaginis cruentus* | KC778969 | KC778818 | KC778948 | KC778840 | KC778862 | KC778927 | KC778906 | KC778884 |
| *Lagopus mutus* | KC785614 | KC785692 | ----------- | KC785643 | KC785659 | KC785676 | KC785709 | KC785726 |
| *Leipoa ocellata* | AF222586 | AF394618 | KC749592 | KC749637 | FJ881843 | KC749734 | AF394647 | KC749880 |
| *Lophophorus impejanus* | KC749458 | DQ307007 | KC749593 | KC749638 | KC749698 | KC749735 | EF569445 | KC749881 |
| *Lophophorus lhuysii* | KC778970 | KC778819 | KC778949 | KC778841 | KC778863 | KC778928 | KC778907 | KC778885 |
| *Lophophorus sclateri* | KC778972 | KC778821 | KC778951 | KC778843 | KC778865 | KC778930 | KC778909 | KC778887 |
| *Lophura inornata* | KC749459 | DQ307008 | KC749594 | KC749639 | KC749699 | KC749736 | EF569446 | KC749882 |
| *Lophura leucomelanos* | KC778971 | KC778820 | KC778950 | KC778842 | KC778864 | KC778929 | KC778908 | KC778886 |
| *Lophura nycthemera* | KC749460 | DQ307009 | KC749595 | KC749640 | FJ881853 | KC749737 | EF569447 | KC749883 |
| *Lophura swinhoii* | KC749461 | DQ307010 | KC749596 | KC749641 | KC749700 | KC749738 | EF569448 | KC749884 |
| *Margaroperdix madagascarensis* | KC785615 | KC785693 | KC785629 | ----------- | KC785660 | KC785677 | KC785710 | KC785727 |
| *Megapodius eremita* | AY274005 | AY274052 | EU738169 | JF496975 | EU738642 | EU739274 | EU737237 | ----------- |
| *Megapodius layardi* | AY952761 | AF394635 | KC749598 | KC749643 | FJ881844 | KC749740 | AF394657 | KC749886 |
| *Meleagris gallopavo* | U83741 | AF222556 | KC749597 | KC749642 | FJ881856 | KC749739 | AY952758 | KC749885 |
| *Numida meleagris* | AF222587 | AF394613 | KC749599 | KC749644 | KC749701 | KC749741 | AF394642 | KC749887 |
| *Oreortyx pictus* | AY952765 | AY952749 | KC749600 | KC749645 | KC791426 | KC749742 | AY952755 | KC749888 |
| *Ortalis vetula* | AY952762 | AF394614 | KC749601 | KC749646 | FJ881846 | KC749743 | AY952751 | KC749889 |
| *Oxyura jamaicensis* | AY747700 | EU585721 | EU738184 | JF496988 | EU738657 | EU739288 | EU737253 | ----------- |
| *Pavo cristatus* | AY952766 | AF394612 | KC749602 | KC749647 | KC749702 | KC749744 | AF394640 | KC749890 |
| *Pavo muticus* | KC749462 | EF569478 | KC749603 | KC749648 | KC749703 | KC749745 | EF569449 | KC749891 |
| *Perdix dauurica* | KC785616 | KC785694 | KC785630 | KC785644 | KC785661 | KC785678 | KC785711 | KC785728 |
| *Perdix hodgsoniae* | KC778973 | KC778822 | KC778952 | KC778844 | KC778866 | KC778931 | KC778910 | KC778888 |
| *Perdix perdix* | KC749469 | AF222560 | KC749611 | KC749656 | FJ881852 | KC749753 | EF569456 | KC749899 |
| *Phasianus colchicus* | U83742 | AF222561 | KC749608 | KC749653 | FJ881851 | KC749746 | AY952759 | KC749896 |
| *Polyplectron bicalcaratum* | KC749463 | EF569479 | KC749604 | KC749649 | KC749704 | KC749746 | EF569450 | KC749892 |
| *Polyplectron chalcurum* | KC749464 | EF569480 | KC749605 | KC749650 | KC749705 | KC749747 | EF569451 | KC749893 |
| *Polyplectron germaini* | KC749466 | DQ768266 | KC749607 | KC749652 | KC749706 | KC749751 | EF569453 | KC749895 |
| *Polyplectron inopinatum* | KC749467 | EF569482 | KC749608 | KC749654 | KC749707 | KC749751 | EF569454 | KC749897 |
| *Polyplectron katsumatae* | KC778974 | KC778823 | KC778953 | KC778845 | KC778867 | KC778932 | KC778911 | KC778889 |
| *Polyplectron malacense* | KC749468 | DQ768268 | KC749610 | KC749655 | KC749708 | KC749752 | EF569455 | KC749898 |
| *Polyplectron napoleonis* | KC749465 | EF569481 | KC749606 | KC749651 | FJ881858 | KC749748 | EF569452 | KC749894 |
| *Pternistis ahantensis ** | KC785609 | KC785688 | KC785625 | KC785640 | KC785654 | KC785671 | KC785704 | KC785721 |
| *Pternistis bicalcaratus ** | KC785610 | KC785689 | KC785626 | KC785641 | KC785655 | KC785672 | KC785705 | KC785722 |
| *Pternistis swainsonii ** | KC785613 | KC785691 | KC785628 | ----------- | KC785658 | KC785675 | KC785708 | KC785725 |
| *Pucrasia macrolopha* | KC749470 | DQ768269 | KC749612 | KC749657 | KC749709 | KC749755 | EF569457 | KC749900 |
| *Rollulus rouloul* | KC749471 | KC749859 | EU738207 | JF497007 | EU738688 | KC749756 | EU737280 | KC749901 |
| *Scleroptila africanus ** | KC785608 | KC785687 | ----------- | KC785639 | KC785653 | KC785670 | KC785703 | KC785720 |
| *Syrmaticus ellioti* | KC749472 | DQ307011 | KC749613 | KC749658 | KC749710 | KC749755 | EF569458 | KC749900 |
| *Syrmaticus humiae* | KC778977 | KC778826 | KC778956 | KC778848 | KC778870 | KC778934 | ----------- | KC778892 |
| *Syrmaticus mikado* | KC778978 | KC778827 | KC778957 | KC778849 | KC778871 | KC778935 | KC778914 | KC778893 |
| *Syrmaticus reevesii* | KC749473 | DQ307012 | KC749614 | KC749659 | KC749711 | KC749756 | EF569459 | KC749901 |
| *Tetraogallus altaicus* | KC785617 | KC785695 | KC785631 | KC785645 | KC785662 | KC785679 | KC785712 | KC785729 |
| *Tetraogallus himalayensis* | KC785618 | KC785696 | KC785632 | KC785646 | KC785663 | KC785680 | KC785713 | KC785730 |
| *Tetraogallus tibetanus* | KC778980 | KC778829 | KC778959 | KC778851 | KC778873 | KC778937 | KC778916 | KC778895 |
| *Tetrastes bonasia* | KC778964 | KC778813 | KC778943 | KC778835 | KC778857 | KC778922 | KC778901 | KC778879 |
| *Tetrastes sewerzowi* | KC778979 | KC778828 | KC778958 | KC778850 | KC778872 | KC778936 | KC778915 | KC778894 |
| *Tragopan blythii* | KC749474 | DQ307013 | KC749615 | KC749660 | KC749712 | KC749757 | EF569460 | KC749904 |
| *Tragopan caboti* | KC778981 | KC778830 | ----------- | KC778852 | KC778874 | KC778938 | KC778917 | KC778896 |
| *Tragopan temminckii* | AF222595 | AF222566 | KC749616 | KC749661 | KC749712 | KC749758 | AY952760 | KC749905 |
| *Tympanuchus phasianellus* | KC749475 | AF222569 | KC749617 | KC749662 | KC749714 | KC749759 | EF569461 | KC749906 |

*** *Dendroperdix*, *Pternistes*, and *Scleroptila* are often placed in the genus *Francolinus*, but are not monophyletic (see Crowe et al. 2006); *Excalfactoria* is often referred to as *Coturnix*.
